# Natural Agents for Preventing Skin Damage Induced by Visible Light: A Systematic Review of Preclinical and Clinical Evidence

**DOI:** 10.1111/phpp.70113

**Published:** 2026-08-06

**Authors:** Azahara Rodríguez‐Luna, Alicia Zamarrón, Cristina García‐Muñoz, Juan‐Carlos Hernández‐Rodríguez, Henry W. Lim, Salvador González

**Affiliations:** ^1^ Faculty of Health Sciences Universidad Loyola Andalucía Sevilla Spain; ^2^ Department of Experimental Dermatology and Skin Biology Instituto Ramón y Cajal de Investigación Sanitaria, IRYCIS Madrid Spain; ^3^ Department of Biology, Faculty of Sciences Autonoma University of Madrid (UAM) Madrid Spain; ^4^ Department of Physiotherapy Universidad de Sevilla Sevilla España; ^5^ Department of Dermatology Hospital Universitario Virgen del Rocío Sevilla España; ^6^ The Henry W. Lim Division of Photobiology and Photomedicine, Department of Dermatology Henry Ford Health Detroit Michigan USA; ^7^ Department of Medicine and Medical Specialties Alcalá de Henares University Madrid Spain

**Keywords:** high‐energy visible light, natural compounds, photoaging, photoprotection, pigmentation, skin photobiology, systematic review, visible lightantioxidants

## Abstract

**Background/Purpose:**

Visible light (VL), and particularly high‐energy visible light (HEVL), reaching Earth's surface has emerged as a relevant contributor to skin damage. VL has been implicated in oxidative stress, inflammation, pigmentation disorders, and photoaging, especially in individuals with darker skin phototypes. As conventional sunscreens offer limited protection in the visible spectrum, increasing attention has been directed toward complementary photoprotective strategies, including the use of natural compounds with antioxidant and anti‐inflammatory properties. This systematic review aims to evaluate and synthesize the available preclinical and clinical evidence on the photoprotective effects of natural compounds against VL‐induced skin damage, emphasizing their mechanisms of action, efficacy, and safety.

**Methods:**

A comprehensive literature search was performed to identify studies reporting validated objective outcomes, including colorimetric parameters, diffuse reflectance spectroscopy, or clinical grading scales. Eligible studies were included, qualitatively synthesized, and risk of bias was evaluated.

**Results:**

Preclinical studies demonstrated that natural compounds mitigated VL‐induced oxidative stress, inflammation, and pigmentation‐related pathways. Clinical studies showed that formulations containing natural compounds improved VL‐induced erythema and pigmentation outcomes. However, considerable heterogeneity was observed in irradiation protocols, outcome measures, and study designs, precluding quantitative meta‐analysis.

**Conclusion:**

Overall, available evidence supports a protective role for selected natural compounds against VL‐induced skin damage, particularly through antioxidant and anti‐inflammatory mechanisms, with the strongest clinical evidence focusing on natural sources of polyphenols. Nevertheless, this review highlights the need for standardized phototesting protocols and well‐designed clinical trials. Future research should focus on comparative efficacy, long‐term safety, and integration of natural compounds into combined photoprotection strategies tailored to different skin phototypes.

## 1 Introduction

Visible light (VL, 400–700 nm) accounts for approximately 50% of solar radiation that reaches the Earth's surface and represents the only portion of the electromagnetic spectrum perceptible to the human eye. Beyond natural solar exposure, the “digital age” has introduced chronic contact with artificial light sources, or Electronic Device Generated Light (EDGL), such as Light‐Emitting Diodes (LEDs) and digital screens. These sources primarily emit high‐energy visible light (HEVL) within the 400–500 nm range [1, 2, 3]. While the absolute irradiance of EDGL remains significantly lower than that of solar VL, and although current clinical evidence suggests that its emission levels may be insufficient to independently exacerbate pigmentary conditions such as melasma [4], its chronicity and proximity to the integument have raised concerns regarding its cumulative contribution to the total cutaneous oxidative burden [5].

The deleterious effects of VL are dictated by a complex interplay of wavelength, cumulative dose, and individual susceptibility. Unlike UVR, which is primarily absorbed by DNA and proteins, VL interacts with endogenous chromophores such as melanin, flavins, and porphyrins, as well as specialized photoreceptors like opsin‐3 (OPN3). Recent evidence indicates that OPN3 activation in melanocytes triggers a calcium‐dependent signaling cascade that not only stimulates tyrosinase activity but also inhibits autophagy, thereby decreasing melanosome degradation [6]. This mechanism facilitates sustained neomelanogenesis, explaining why clinical Immediate Pigment Darkening (IPD) and Persistent Pigment Darkening (PPD), which result from the oxidation of pre‐existing melanin, are significantly more intense and long‐lasting in skin phototypes III–VI compared to those induced by UVA [1, 3]. At the subcellular level, HEVL promotes mitochondrial dysfunction and overproduction of reactive oxygen species (ROS) through the excitation of respiratory chain components. This oxidative surge activates redox‐sensitive signaling cascades, including mitogen‐activated protein kinases (MAPK/p38) and nuclear factor kappa B (NF‐κβ), which orchestrate the release of pro‐inflammatory cytokines and the upregulation of matrix metalloproteinases (MMPs). Collectively, these processes drive the degradation of the extracellular matrix (ECM), accelerating extrinsic aging (photoaging) and exacerbating VL‐sensitive photodermatoses and pigmentary disorders, including solar urticaria, cutaneous porphyrias, post‐inflammatory hyperpigmentation (PIH) and melasma [1, 2, 7, 8]. Furthermore, while VL is not directly genotoxic, it impairs nucleotide excision repair (NER) mechanisms and contributes to the formation of dark cyclobutane pyrimidine dimers (dark CPDs) through melanin‐mediated chemiexcitation [5, 9].

Traditional filters provide limited protection against the visible spectrum, as their regulatory efficacy is largely confined to wavelengths below 370 nm. This “spectral gap” leaves the skin vulnerable to VL‐induced oxidative and pigmentary damage. Although tinted formulations incorporating iron oxides act as effective physical barriers against HEVL, physical blockage alone may be insufficient to manage the oxidative cascade triggered by the fraction of radiation that bypasses such filters [10, 11]. Consequently, achieving “spectral homeostasis” requires the integration of secondary biological defenses, such as natural compounds (polyphenols, vitamins, and algal extracts), which act as potent ROS scavengers and anti‐inflammatory modulators.

The incorporation of natural compounds, particularly botanically derived polyphenols, vitamins, and algal extracts, represents a promising frontier in expanding the photoprotective armamentarium. Unlike traditional filters, these agents offer a multi‐targeted approach, acting as potent ROS scavengers, anti‐inflammatory modulators, and genomic stabilizers. However, despite the growing interest in natural photoprotection, current evidence remains fragmented [12, 13, 14].

To address this important gap in the existing literature, this systematic review aims to critically appraise and synthesize the available evidence on the photoprotective effects of natural compounds against VL‐induced skin damage. This review will move beyond a simple descriptive analysis by emphasizing the precise molecular mechanisms of action, evaluating clinical efficacy across various skin phototypes, and identifying the methodological limitations that currently hinder the standardization and integration of natural agents within modern dermatological therapy.

## 2 Methods

The systematic review protocol was prospectively registered in the PROSPERO database (CRD420251116264). This systematic review was conducted in accordance with the PRISMA (Preferred Reporting Items for Systematic Reviews and Meta‐Analyses) 2020 guidelines [15]. Although the initial study protocol intended to perform a meta‐analysis, the substantial lack of reported numerical data (such as means, standard deviations, or exact effect sizes) across the included studies precluded a quantitative synthesis. Consequently, a systematic narrative synthesis was performed. To ensure the robustness of this qualitative approach, the methodological quality and risk of bias were rigorously evaluated using specific validated tools tailored to each study design: ROB‐2 for randomized controlled trials, ROBINS‐I for non‐randomized clinical studies, and the OHAT approach for preclinical evidence. During the preparation of this manuscript, the authors used Gemini for text processing and for the generation of the graphical abstract. The authors reviewed and edited the content as needed and take full responsibility for the integrity of the manuscript. These tools were used only as technical aids and are not listed as authors.

### 2.1 Data Sources and Search Strategy

A systematic literature search was conducted in PubMed/MEDLINE, Scopus, Web of Science, and the Cochrane Library, without date restrictions from inception to 2025 by reviewers A.R.‐L. and A.Z. The search strategy combined terms related to VL exposure, skin damage, photoprotection, and natural compounds. Manual citation searching related to our topic studies were also performed to identify additional potential studies. The detailed search strategy and selection process are reported in File S1.

### 2.2 Research Question and Eligibility Criteria

The research question was formulated using the PICOS (Population, Intervention, Comparator, Outcome and Study design) framework, adapted to experimental and clinical evidence [16].

#### 2.2.1 Population

- *Preclinical studies*: Human skin–relevant cellular models, including human keratinocytes, fibroblasts, melanocytes, reconstructed human epidermis, and human skin explants exposed to VL, HEV/blue light (BL), and/or VL + UVA1.
- *Clinical studies*: Healthy human volunteers undergoing controlled phototesting with VL, HEV/BL, and/or VL + UVA1.

#### 2.2.2 Interventions

Interventions involving natural compounds or formulations containing natural ingredients with proposed photoprotective properties, administered topically or orally. These encompassed botanical extracts, isolated phytochemicals, antioxidant blends, and natural compound–enriched sunscreens.

#### 2.2.3 Comparators

Comparators included untreated controls (even intra‐subject), placebo or vehicle controls, non‐antioxidant sunscreen formulations, or baseline non‐irradiated conditions.

#### 2.2.4 Outcomes

Eligible studies must report at least one quantitative or semi‐quantitative outcome relevant to VL‐induced skin responses:

- Preclinical outcomes:
  ○ Cellular viability and morphology
  ○ Oxidative stress markers (e.g., ROS, malondialdehyde (MDA), superoxide dismutase (SOD))
  ○ Mitochondrial function and membrane potential
  ○ Inflammatory and signaling pathways (e.g., MAPK, p38, Nrf2, OPN3)
  ○ Pigmentation‐related outcomes (melanin synthesis, melanogenic signaling)
- Clinical outcomes:
  ○ Colorimetric parameters (L*, a*, b*, Δ*a*, ΔL*, ITA°, ΔITA, Δ*E*)
  ○ Diffuse reflectance spectroscopy (DRS) parameters (erythema, melanin content, AUC 400–700 nm)
  ○ Validated clinical grading scales (e.g., Investigator's Global Assessment for erythema and pigmentation)

#### 2.2.5 Study Design

Only interventional studies were included, comprising controlled in vitro/ex vivo experiments and controlled clinical phototesting trials.

### 2.3 Exclusion Criteria

Studies were excluded based on the following criteria:

- Studies exclusively conducted in animal models without direct relevance to human skin photoprotection.
- Studies that only focus on plant biology, phytochemical biosynthesis, or callus cultures without evaluating photoprotection in skin‐relevant models.
- Studies that use non‐cutaneous cell lines (e.g., ocular, neuronal, or cancer models) unrelated to skin photobiology.
- Study protocols, theses, dissertations, and conference proceedings.
- Studies lacking validated or reproducible outcome measures.

### 2.4 Study Selection

Two reviewers (A.R.‐L. and A.Z.) independently evaluated all retrieved records for eligibility. In the initial screening phase, titles and abstracts were reviewed to identify potentially relevant studies, and duplicate records were removed using a structured Microsoft Excel spreadsheet (Microsoft Corp.). Full‐text articles meeting the preliminary inclusion criteria were subsequently imported into Mendeley desktop (version 2.132.1) reference management software (and independently assessed in full by the same two reviewers, A.R.‐L. and A.Z.) meeting the predefined eligibility criteria. Any discrepancies between reviewers at this stage were resolved through discussion and consensus, with arbitration by a third reviewer (S.G.) when necessary.

### 2.5 Data Extraction

Data were gathered using a standardized and predefined extraction form developed in Microsoft Excel (Microsoft Corp. Redmond, WA, USA). Data extraction was performed independently by two reviewers (A.R.‐L. and A.Z.). Any discrepancies during the process were resolved through discussion and consensus between the two researchers; in cases where agreement could not be reached, a third reviewer (S.G.) acted as an arbitrator to make the final decision. For preclinical studies, extracted data included the cell or tissue model employed, characteristics of the natural compound and its dosing protocol, irradiation spectrum and dosage, intervention and comparison groups and reported outcome measures. These included mechanistic endpoints related to cytotoxicity, DNA damage, oxidative stress responses, inflammatory responses, pigmentation changes, and signaling pathway alterations.

For clinical research studies, extracted variables comprised characteristics of the natural compound and dosing regimen details, irradiation protocol parameters (wavelength range, dose, and irradiance), participant demographics (sample size, age, sex, skin phototype, and ethnicity), characteristics of the intervention and comparison groups, and reported clinical outcomes related to erythema, pigmentation, and histological outcomes.

When information was incomplete or presented exclusively in graphical format, this limitation was explicitly recorded in the extraction table. No assumptions were made regarding missing data.

### 2.6 Risk of Bias Assessment

Risk of bias was independently assessed according to the study design using validated tools. Randomized controlled trials were evaluated using the Cochrane Risk of Bias tool version 2 (ROB‐2), which considers bias arising from the randomization process, deviations from intended interventions, missing outcome data, outcome measurement, and selective reporting. Non‐randomized clinical studies were assessed using the Risk Of Bias In Non‐Randomized Studies‐of Interventions (ROBINS‐I) tool, covering bias due to confounding, participant selection, intervention classification, deviations from intended interventions, missing data, outcome measurement, and selective reporting.

Preclinical in vitro and ex vivo studies were evaluated using the Office of Health Assessment and Translation (OHAT) risk‐of‐bias tool, adapted for experimental laboratory research. This tool addresses randomization, allocation concealment, blinding, confounding, exposure characterization, outcome assessment, incomplete data, selective reporting, conflicts of interest, and other sources of bias, with judgments categorized as definitely low, probably low, probably high, or definitely high risk of bias.

Two independent reviewers (JCHR and CGM) examined the risk of bias using the mentioned tools. The percentage of agreement between reviewers was calculated for all the tools. Any discrepancies between reviewers were resolved by consensus.

### 2.7 Outcome Harmonization and Data Synthesis

Given the substantial heterogeneity observed across included studies with respect to experimental models, irradiation protocols, outcome measures, and reporting formats, results were synthesized using a qualitative narrative approach. Evidence from preclinical and clinical studies was analyzed and reported separately and summarized in structured evidence tables. These tables were further stratified according to study type (preclinical versus clinical), light spectrum evaluated (VL, HEV/BL, or combined VL and UVA1 exposure), and the type of natural compound or formulation assessed.

A quantitative meta‐analysis was not undertaken due to several methodological constraints, including the frequent absence of numerical outcome data (e.g., means and standard deviations), variability in outcome definitions and assessment time points, and marked heterogeneity in irradiation conditions and intervention formulations across the studies.

### 2.8 Certainty of Evidence

The certainty of evidence for clinical outcomes was assessed using the GRADE framework. Outcomes related to visible light–induced skin damage, including pigmentation, erythema, and objective biophysical or molecular markers, were considered critical. Randomized controlled trials were initially rated as high certainty and non‐randomized studies as low certainty. Ratings were subsequently modified according to risk of bias, inconsistency, indirectness, imprecision, and publication bias. Risk of bias was informed by ROB‐2 and ROBINS‐I assessments. Inconsistency and indirectness were evaluated considering heterogeneity in irradiation protocols, outcome definitions, and clinical relevance. Imprecision was judged qualitatively based on sample size and robustness of effects, given the absence of meta‐analysis. Publication bias was explored qualitatively due to the limited number of studies. According to GRADE methodology, preclinical in vitro and ex vivo studies were not included in certainty assessments, as the framework is intended for clinical decision‐making based on human evidence; such studies were used only to support biological plausibility. All assessments were performed independently by two reviewers (J‐C.H‐R and C G‐M) with consensus resolution (S.G).

## 3 Results

### 3.1 Study Characteristics

**FIGURE 1:**
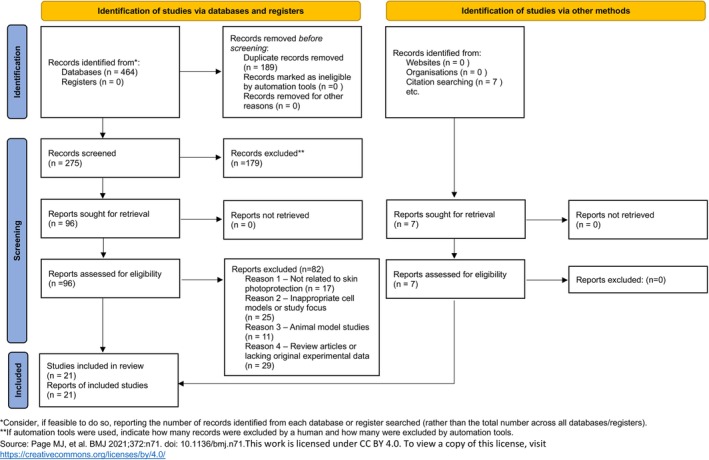
PRISMA 2020 flow diagram illustrating the study identification, screening, eligibility assessment, and inclusion process for the present systematic review, including records identified through database searches, registers, and other sources. Adapted from [17], under the Creative Commons Attribution 4.0 International (CC BY 4.0) license.

Following an initial screening of titles and abstracts, and subsequent full‐text review according to the predefined eligibility criteria, the studies meeting inclusion criteria were retained for qualitative synthesis (Figure 1). The included articles were published between 2008 and 2024 and encompassed both preclinical and clinical investigations evaluating photoprotective strategies against VL, HEV/BL, and/or VL + UVA1 exposure.

Based on the nature of the intervention, the studies were categorized according to the type of natural compound or formulation evaluated. These categories included Polyphenolic compounds, Polysaccharides and amino‐acid derivatives, Vitamins and carotenoids, and Antioxidant blends, algal extracts and others.

Regarding study design, the evidence base comprised 15 preclinical studies, which employed human skin cell models, human epidermal equivalents or living skin explants, and murine models, and clinical studies involving controlled phototesting trials in human volunteers. Preclinical and clinical characteristics of the included studies are summarized separately in Table 1 (preclinical studies) and Table 2 (clinical studies), while the overall.

**TABLE 1:** Summary of preclinical evidence on the photoprotective effects of natural compounds against VL.

Study	Cellular model	Ingredients/Doses	Irradiation/Doses	Intervention groups (IG)	Comparison groups (CG)	Outcome (measures)	Main results
Botta et al. (2008)	Human keratinocytes	Ectoin (0.025, 0.05, 0.1 mM) (1 h, 37°C) L‐ergothioneine (ERT) (0.1, 0.25, 0.5 mM) Mannitol (0.5, 1, 1.5 mM) Tinted and not‐tinted sunscreens—Photoderm SPF 50 + , tinted Photoderm SPF 50 + , Photoderm Max SPF100 and tinted Photoderm Max SPF100 (2 mg/cm^2^)	UVA/VL: 315–800 nm (8% UVA, 92% VL); doses 5, 10, 15 J/cm^2^ VL alone: 400–800 nm; doses 5, 10, 15 J/cm^2^	IG1: Keratinocytes incubated with compounds (ectoin, ERT or mannitol) and exposed to UVA/VL or VL IG2: Keratinocytes incubated with sunscreens and exposed to UVA/VL or VL	Non‐irradiated cells (negative control) Non‐irradiated cells treated with MMS (internal DNA‐damage control for comet assay) Irradiated keratinocytes/CHO cells without any protective treatment (positive control) Non‐treated, non‐irradiated keratinocytes (baseline control for protective‐effect assays)	Cell viability (WST‐1 assay) DNA damage (comet assay, OTM χ^2^ values) Photoprotection by ectoin, ERT, mannitol, and sunscreens (comet assay)	*DNA damage (comet assay)*: 5 J/cm^2^: OTM χ^2^ 4.78 ± 0.79 for UVA/VL and 3.78 ± 1.00 for VL (vs 2.90 ± 1.25 for non‐irradiated cells). 10 J/cm2: OTM χ2 7.76 ± 1.16 for UVA/VL and 6.34 ± 1.39. 15 J/cm2: OTM χ2 9.37 ± 0.31 79 for UVA/VL and 5.91 ± 0.21 for VL. Protective effect of ectoin, ERT, and mannitol: (Values correspond to highest protective dose tested) Ectoin (0.1 mM): VL: OTM 2.37 ± 0.41 → 92.7% protection UVA/VL: OTM 4.04 ± 0.16 → 68.9% protection L‐ergothioneine (0.5 mM): VL: OTM 2.29 ± 0.19 → 97.9% protection UVA/VL: OTM 5.15 ± 0.20 → 59.8% protection Mannitol (1.5 mM): VL: OTM 5.18 ± 0.19 → 52% protection UVA/VL: OTM 6.71 ± 0.29 → 62.7% protection. Protective capacity of sunscreens: Photoderm SPF 50 + : UVA/VL: OTM 3.64 ± 0.23 → 79.9% protection VL: OTM 2.77 ± 0.24 → 82.8% protection. Photoderm Max SPF100: UVA/VL: OTM 3.14 ± 0.15 → 86.7% protection VL: OTM 2.47 ± 0.24 → 92.5% protection Tinted Photoderm SPF 50 + : UVA/VL: OTM 2.69 ± 0.22 → 92.6% protection VL: OTM 2.12 ± 0.08 → 100% protection Tinted Photoderm Max SPF100: UVA/VL: OTM 2.40 ± 0.21 → 97.1% protection VL: OTM 2.16 ± 0.12 → 100% protection
Liebel et al. (2012)	Primary human keratinocytes Human epidermal equivalents.	Antioxidant blend composed of Feverfew ( *Tanacetum parthenium* ) extract + Soy ( *Glycine soja* ) extract + Gamma Tocopherol UVA/UVB sunscreen	VL: 65, 130, 180 and 240 J/cm^2^	IG1: Human skin epidermal equivalents treated with antioxidant blend and exposed to VL IG2: Human skin epidermal equivalents treated with UVA/UVB sunscreen and exposed to VL IG3: Human skin epidermal equivalents treated with antioxidant blend+UVA/UVB sunscreen and exposed to VL	CG1: Human primary keratinocytes/equivalents exposed to VL (65–180 J/cm^2^) (positive control) CG2: Non‐irradiated cells/equivalents (negative control) CG3: Human primary keratinocytes treated with TNFα (positive control) (for EGFR–p42/44 MAPK analysis)	ROS production in epidermal equivalents Release of proinflammatory cytokines (L‐1α, IL‐6, GM‐CSF, IL‐8, TNFα, MMP‐1 and MMP‐9) in epidermal equivalents (ELISA) EGFR–p42/44 MAPK pathway alteration in human primary keratinocytes (Western blotting) Thymine dimer formation in epidermal equivalents (T–T dimer staining)	IG1: Pretreatment with antioxidants similarly reduces ROS, IL‐1α, and MMP‐1 release IG2: Pretreatment with UV sunscreen alone had no protective effects against VL IG3: Pretreatment with antioxidants + UV‐sunscreen reduces ROS, IL‐1a, and MMP‐1 release by 78%, 82%, and 87%, respectively (*No numeric values reported*)
Avola et al. (2019)	‐Human keratinocytes (NCTC 2544) ‐Human dermal fibroblasts	Hydroxytyrosol (from *Olea europaea* fruits) (10, 25, and 50 μg/mL)	Blue LED light (LED‐BL):5–85 J/cm^2^	Keratinocytes and fibroblasts pretreated with hydroxytyrosol (at 10, 25, and 50 μg/mL) for 24 h, followed by LED‐BL exposure	CG1: Untreated, non‐irradiated cells (negative control) CG2: Untreated cells exposed to LED‐BL (positive control)	Cell viability (MTT assay) ROS intracellular production (H2‐DCFDA assay) DNA damage (8‐hydroxy‐2′‐deoxyguanosine quantification) Expression of collagen I, MMP‐1 and MMP‐12 (RT‐PCR and Western blotting) Expression of PCNA (Western blotting and immunocytochemistry)	*Cell viability*: Hydroxytyrosol showed no cytotoxicity up to 250 μg/mL and preserved cell viability against LED‐BL in both cell types *ROS intracellular production*: LED‐BL markedly increased ROS levels in keratinocytes and fibroblasts; hydroxytyrosol pretreatment significantly attenuated ROS formation *DNA damage*: LED‐BL at 15 and 45 J/cm^2^ strongly increased 8‐OHdG levels; hydroxytyrosol reduced this damage in a dose‐dependent manner *Biomarkers' expression*: LED‐BL increased MMP‐1 and MMP‐12 and reduced collagen I expression; hydroxytyrosol counteracted these effects in a dose‐dependent manner. LED‐BL also increased PCNA expression, which was inhibited by hydroxytyrosol (*No numeric values reported*)
Zamarrón et al. (2018)	Human dermal fibroblasts	Fernblock^(R)^ (FB) (0.5 and 1 mg/mL)	VL: 61.8–247.3 J/cm^2^	IG1: Cells pretreated with FB (0.5 or 1 mg/mL) for 24 h and subsequently exposed to VL	CG1: Untreated, non‐irradiated cells (negative control) CG2: Cells exposed to VL without FB pretreatment (positive control)	Cell morphology (microscopy imaging) Cell viability (flow cytometry, FACS) Cell cycle (flow cytometry) ECM proteins expression (MMP‐1, CTSK, FBN1/2 and ELN) (RT‐PCR and/or immunofluorescence)	*Cell morphology*: VL induces cytoplasmic retraction and cellular stretching. Pretreatment with FB delayed the appearance of these morphological alterations *Cell viability*: FB pretreatment reduced cell death rates to levels comparable to controls *Cell cycle*: VL alone and FB + VL increased the proportion of cells in S and G2/M phases and decreased G0/G1 phase population *ECM proteins expression*: MMP1: increased after VL; pretreatment with FB inhibited this increase. CTSK: increased after VL; FB0.5 mg/mL inhibited this increase. FBN1: increased after VL and FB + VL FBN2: increased after VL; FB 0.5 mg/mL inhibited this increase. ELN: increased after VL and FB + VL (*No numeric values reported*)
Freitas et al. (2019)	Murine melanoma B16‐F10 melanocytes	Apigenin (API) Chrysin (CRI) beta‐carotene (BTC) Dosage: 6.8–100 μg/mL	VL: 12–36 J/cm^2^ (experimental groups irradiated at 36 J/cm^2^)	IG1: B16‐F10 cells induced for melanogenesis (1 mM tyrosine +2.5 mM NH₄Cl), treated with API, CRI, or BTC (6.8–100 μg/mL) for 24 h, followed by VL exposure (36 J/cm^2^).	CG1: B16‐F10 exposed to VL without antioxidant treatment (positive control) B16‐F10 cells treated with antioxidants but not exposed to VL (treatment control)	Cell viability (MTT assay) Singlet oxygen detection (spectrophotometry) Membrane photoprotection	*Cell viability*: API at 6.8–100.0 μg/mL suppressed VL‐induced phototoxic effect (16% photoprotection at 6.8 μg/mL) CRI conferred photoprotection ability at 6.8 μg/mL, whereas higher doses decreased viability BTC increased cell survival by roughly 10% after VL (at 6.8, 46.4, 68.1 and 100.0 μg (mL)) *Singlet oxygen detection*: VL‐induced melanin excitation generated singlet oxygen in B16‐F10 cells; all API, CRI and BTC suppressed singlet oxygen formation *Membrane photoprotection*: API provided significant membrane protection against VL‐induced damage, whereas CRI and BTC did not. (*No numeric values reported*)
Mann et al. (2020)	Primary human dermal fibroblasts	Licochalcone A (LicA): Solution of 0.1% LicA‐rich licorice extract in DMSO Sunscreen (SPF 50+/UVA‐PF 40) containing 0.025% licorice extract (0.005% LicA)	VL: 150 mJ/cm^2^ (> 400 nm, > 450 nm, > 500 nm, and > 585 nm)	IG1: Fibroblasts pretreated with LicA (1 μmol/L, 24 h) and subsequently exposed to VL	CG: Unirradiated fibroblasts (negative control)	ROS production (DCF H2DCFDA assay) Nrf2 activation assay	*ROS production*: VL in the 400 to 450 nm range inducedthe highest ROS levels, with progressively lower ROS generation at longer wavelengths. Pretreatment with LicA at 0.25–2.0 μmol/L markedly reduced VL‐induced ROS formation. *Nrf2 activation assay*: LicA treatment enhanced Nrf2 activation, producing a five‐fold increase relative to controls (*No numeric values reported*)
Lorrio et al. (2020)	Human dermal fibroblasts (HDF) and melanocytes	*Deschampsia Antarctica* extract (EDA, Edafence^(R)^) (0.1–0.3 mg/mL)	BL: 38, 76 and 151 J/cm^2^	IG1: Fibroblasts pretreated with EDA for 24 h and subjected to BL IG2: Melanocytes pretreated with EDA for 24 h and subjected to BL	CG1 (fibroblasts): Fibroblasts not treated with EDA and not exposed to BL (negative control); fibroblasts exposed to BL without EDA pretreatment (positive control) CG2 (melanocytes): Melanocytes not treated with EDA and not exposed to BL (negative control); melanocytes exposed to BL without EDA pretreatment (positive control)	Cell viability (MTT assay) ROS production (H_2_DHFDA assay) Mitochondrial morphology (MitoTracker) Mitochondrial membrane potential (JC‐1 marker) Pigmentation outcomes: p38 melanogenic pathway activation in fibroblasts (Western blotting) intra/extracellular melanin content in melanocytes	*Cell viability*: BL reduced cell viability in both fibroblasts and melanocytes IG1: EDA 0.1 mg/mL significantly increased fibroblasts viability after 38 J/cm^2^ BL and produced a modest protective effect at 76 J/cm^2^ IG2: EDA 0.1 mg/mL induced a modest increase of viability in BL‐exposed melanocytes. *ROS production*: BL increased intracellular ROS in fibroblasts, whereas EDA pretreatment (0.1 mg/mL) reduced BL‐induced ROS levels *Mitochondrial morphology*: BL altered mitochondria morphology (shorting, round‐shaping, granulation) EDA preserved mitochondrial alterations induced by BL *Mitochondrial membrane potential*: BL induced membrane hyperpolarization; EDA prevented this BL‐induced alteration *Pigmentation*: BL activated the p38 melanogenic pathway (in fibroblasts), whereas EDA reduced p38 phosphorylation. BL increased both intracellular and extracellular melanin content in melanocytes; EDA attenuated BL‐induced hyperpigmentation (*No numeric values reported*)
Ayres et al. (2021)	Human melanocytes HEMa‐LP	French maritime pine bark ( *‐ Pinus pinaster* ) extract (PBE) (Pycnogenol) Doses: 0.0316, 0.0100, and 0.00316 mg/mL.	VL: 480 J/cm^2^ ASS (Association of solar‐simulated radiation): UVA/UVB (10 J/cm^2^) + IR‐A (360 J/cm^2^) + VL (480 J/cm^2^)	IG: Melanocytes treated with PBE and exposed to VL or ASS after 48 h	CG1: Melanocytes not treated with PBE and not exposed to VL/ASS (negative control) Melanocytes only exposed to VL/ASS without PBE pretreatment (positive control)	Melanin synthesis (spectrophotometry) Tyrosinase activity (monophenolase activity assay) Endothelin‐1 (ED1) and peroxisome proliferator‐activated receptors (PPAR) *α*, *δ*, and *γ* production (ELISA)	*Melanin synthesis*: VL increased melanin by 5.98% (*p* < 0.001); ASS increased melanin by 10.56% (*p* < 0.001). PBE did not prevent VL‐induced melanogenesis but effectively inhibited ASS‐induced increases in melanin production. *Tyrosinase activity*: PBE reduced tyrosinase activity, with inhibition up to 66.5%. *No SD values reported*. *ED1 production*: VL and ASS induced overproduction of ED1 (VL caused an increase of 63.81% and ASS of 57.81%, *p* < 0.001) PBE pretreatment reduced ED1 overproduction after VL (58.02% reduction at 0.0316 mg/mL; 31.92% at 0.0100 mg/mL) and after ASS (up to 73.05% reduction at 0.0100 mg/mL) (*no SD values reported*) *PPAR α, δ, and γ production*: VL did not significantly upregulate PPARs. ASS significantly increased PPAR *α*, *δ*, and *γ*. PBE pretreatment prevented these ASS‐induced increases, reducing levels by 56.60% across all tested concentrations (*no numeric SD values reported*)
Pressi et al. (2021)	Human skin keratinocytes (HaCaT). Human living skin explants (SPT II‐III)	*Melissa officinalis* dried powder phytocomplex with high content of rosmarinic acid and polyphenols. Doses: 0.1% for cell cultures 0.1% and 0.05% w/v topically applied (2 mg/cm^2^) for explants	BL: 63.75 J/cm^2^	IG1: HaCaT exposed to H_2_O_2_ and treated with *Melissa officinalis* phytocomplex IG2: Human skin explants treated with *Melissa officinalis* phytocomplex (daily application for 5 days) and subsequently exposed to BL	CG1: Untreated HaCaT cells (negative control). CG2: HaCaT cells treated with N‐acetyl‐L‐cysteine or rutin (positive antioxidant controls). CG3: Unirradiated explants (baseline control)	ROS levels in HaCaT keratinocytes (DCF‐DA assay) BL‐induced damage and protective effects in skin explants: ○ Cell viability ○ Nrf2 expression (immunostaining)	*ROS levels*: *Melissa Officinalis* phythocomplex inhibited H_2_O_2_‐induced ROS overproduction *Cell viability (skin explants)*: BL exposure did not alter cell viability, and the phytocomplex also did not induce cytotoxicity (no numeric values reported) *Nrf2 expression (skin explants)*: BL increased activated Nrf2 in the epidermis. *Melissa officinalis* phytocomplex reduced this activation at 0.05% and fully suppressed it at 0.1%
Portillo et al. (2021)	Human fibroblasts (HDF) Murine melanocytes (B16‐F10)	Fernblock^(R)^ (FB). Doses: 0.1, 0.3, and 0.5 mg/mL (24 h pretreatment)	BL: 25–151 J/cm^2^.	IG1 (HDF): Fibroblasts pretreated with FB (0.1–0.5 mg/mL) for 24 h and subsequently exposed to BL IG2 (melanocytes): B16‐F10 melanocytes pretreated with FB (0.1–0.5 mg/mL) for 24 h and exposed to BL; additional DHICA oxidation assay performed with FB and ascorbic acid	CG1: Cells without any treatment (negative control). CG2: Cells treated with FB but not‐exposed to BL (treatment control). CG3: Cells exposed to BL without FB pretreatment (positive control) Ascorbic acid as positive antioxidant control in oxidation DHICA test (melanocytes only)	*HDF*: Cell viability (MTT) ROS production (H_2_DCFDA) Mitochondrial membrane potential (JC‐1) MAPK‐p38 expression (Western blot) *Melanocytes*: Opsin‐3 expression (RT‐PCR) Intracellular and extracellular melanin production (spectrophotometry) Melanin oxidation (DHICA test)	*Cell viability in HDF*: BL reduced fibroblast viability; FB at 0.5 mg/mL conferred protection even at 76 J/cm^2^ *ROS production in HDF*: Pretreatment with FB 0.5 significantly reduced the production of ROS *Mitochondrial morphology and membrane potential in HDF*: BL induced mitochondrial hyperpolarization (increased in JC‐1 ratio). FB pretreatment restored JC‐1 ratio, preventing hyperpolarization *Expression of MAPK‐p38 in HDF*: BL increased p38 phosphorilation. FB pretreatment significantly reduced BL‐induced p38 activation *Opsin‐3 activation (melanocytes)*: BL upregulated Opsin‐3 expression; FB pretreatment reduced expression to baseline levels *Melanin production (melanocytes)*: BL induced intra‐ and extracellular hyperpigmentation; FB at 0.5 mg/mL significantly reduced melanin production *Melanin oxidation (melanocytes)*: FB (0.1 and 0.5) demonstrated a protective effect against DHICA‐melanin oxidation, comparable to antioxidant control. (*No numeric values reported*)
Park, Park, et al. (2022)	Human dermal fibroblasts (HDFs)	*Caesalpinia sappan* extract (CSE): 1–1000 μg/mL for antioxidant evaluation 0.313–1.25 μg/mL for skin aging factors evaluation. Brazilin: 0.1 to 1.0 μg/mL	BL: 30 J/cm2 for 1 h 7 min	IG1: HDFs pretreated with *CSE* (1–1000 μg/mL for antioxidant evaluation; 0.313–1.25 μg/mL for aging‐factor assays) for 24 h and subsequently exposed to BL IG2: HDFs pretreated with brazilin (0.1–1.0 μg/mL) for 24 h and subsequently exposed to BL	CG1 (for IG1‐CSE): HDFs not treated with CSE and not exposed to BL (negative control) HDFs exposed to BL without CSE pretreatment (positive control) HDFs treated with CSE but not exposed to BL (treatment control) CG2 (for IG2—brazilin): HDFs not treated with brazilin and not exposed to BL (negative control) HDFs exposed to BL without brazilin pretreatment (positive control) HDFs treated with brazilin but not exposed to BL (treatment control).	Antioxidant ability (SOD‐like activity and DPPH scavenging activity) mRNA expression ofMMP‐1, MMP‐3, collagen type I and collagen type 3 (rt‐PCR) ROS production (DCFDA assay)	*Antioxidant ability*: CSE and brazilin significantly increased SOD‐like activity and DPPH radical scavenging capacity in a dose‐dependent manner, counteracting BL‐induced oxidative stress *No numeric values reported*. *Skin aging factors expression*: Pretreatment with CSE or brazilin inhibited BL‐induced upregulation of MMP‐1 and MMP‐3 mRNA. Both treatments also mitigated the BL‐induced reduction in collagen type I and collagen type III mRNA expression *No numeric values reported*. *ROS production*: CSE pretreatment attenuated BL‐induced ROS generation in HDFs (*No numeric values reported*)
Park, Kim, et al. (2022)	HaCaT human keratinocyte cell line	Yellow chaste weed (YCW) (*Helichrysum arenarium*) extract. Doses: 0.01%, 0.05% and 0.1% Apigenin. Doses: 10, 20, and 30 μM Galangin: 1, 10, and 20 μM *Apigenin and galangin are active components of YWC	BL: 13.68 J/cm^2^	IG1 (YCW): HaCaT cells pretreated with YCW extract (0.01%–0.1%) for 24 h, exposed to BL, and treated again with YCW for 24 h IG2 (Apigenin): HaCaT cells pretreated with apigenin (10–30 μM) for 24 h, exposed to BL, and treated again with apigenin for 24 h IG3 (Galangin): HaCaT cells pretreated with galangin (1–20 μM) for 24 h, exposed to BL, and treated again with galangin for 24 h	HaCaT cells without any treatment (negative control) HaCaT cells exposed to BL without pretreatment (positive control) HaCaT cells pretreated with each tested compound (YCW, apigenin, or galangin) but not irradiated (compound‐specific treatment controls)	Cell cytotoxicity (CCK‐8) Cell proliferation analysis (CellTiter‐Glo, BrdU ELISA, Click‐iT EdU Imaging) Apoptosis (annexin V flow cytometry) Intracellular calcium influx (Fluo‐4 NW assay) ROS production (DCF‐DA assay) Protein expression: TRPV1, JNK, ERK, p38, FOXO3, Lamin B1, AKT, MST 1/2, nCLU, sCLU, Bax and Bcl‐2 (Western blotting)	*Cell cytotoxicity*: Apigenin showed cytotoxicity at 40 μM; galangin at 30 μM *Cell proliferation*: YCW extract, apigenin, and galangin increased proliferation in BL‐irradiated HaCaT *Apoptosis*: BL reduced viable cells to 81.19%; apigenin and galangin increased survival in BL‐irradiated cells. *Intracellular calcium influx*: BL increased Ca^2+^ influx via TRPV1 activation. YCW, apigenin, and galangin reduced TRPV1‐mediated Ca^2+^ influx *ROS production*: BL induced ROS overproduction. Apigenin and galangin significantly reduced ROS levels *Biomarkers' expression*: Apigenin and galangin counteracted BL‐induced MAPK signaling changes (↓JNK, ↓ERK/p38 phosphorylation; ↑FoxO3 phosphorylation; ↓MST1/2 phosphorylation; ↑Akt phosphorylation). They also modulated apoptosis/survival markers (↓nCLU, ↑sCLU, ↓Bax, ↑Bcl‐2). (*No numeric values reported*)
Doyeong et al. (2023)	Human dermal fibroblasts	*Curcuma longa* L. extract (10–200 μg/mL)	BL: 150 J/cm^2^	IG1— *Curcuma longa* extract: Fibroblasts treated with *C. longa* extract (10–200 μg/mL) and subsequently exposed to BL (150 J/cm^2^).	CG1: Fibroblasts without any treatment (negative control) CG2: Fibroblasts exposed to BL without treatment of *C. longa L*.(positive control for BL damage) CG3: Fibroblasts treated with extract but not irradiated (treatment control)	Antioxidant ability (DPPH assay) Reducing power (spectrophotometry) Cytotoxicity of *C. longa* (MTT assay)	*Scavenging ability and reducing power*: *Curcuma longa* → RC_50_ = 40.12 ± 3.07; EC_50_ → 3087.67 ± 44.78 *Cytotoxicity of C. longa*: At 200 μg/mL enhanced the viability of fibroblasts by 38%. (*No numeric values reported*)
Lee and Kim (2023)	Human skin keratinocytes (HaCaT cells)	Anthocyanin antioxidants from cherry fruits (*Prunus serrulata L*): Cyanidin‐3‐O‐glucoside (C3OG) (10–200 μM) Cyanidin‐3‐Orutinoside (C3OR) (10–200 μM)	BL: 20,000 lx for 1 h	IG1—C3OG: HaCaT cells pretreated with C3OG (10–200 μM) for 24 h and exposed to BL IG2—C3OR: HaCaT cells pretreated with C3OR (10–200 μM) for 24 h and exposed to BL	CG1: HaCat without any treatment (negative control) CG2: HaCaT exposed to BL without C3OG/C3OR pretreatment (positive control) CG3: HaCaT pretreated with C3OG or C3OR but not irradiated (compound‐specific treatment controls) CG4:NAC (a ROS scavenger) (positive control for ROS production assay)	Citotoxicity (MTT assay) ROS production (H2DCFDA assay) MAPK activity (Immunoblotting for FAK, MAPKs, IKKα) Pro‐inflammatory cytokine production (TNF‐α, IL‐6, IL‐8; ELISA) Apoptosis (caspase‐3 activation; cleavage of FAK and PARP; Immunoblotting)	*Cytotoxicity*: C3OG and C3OR reversed BL‐induced cytotoxicity in HaCaT. *ROS production*: Pre‐treatment with either C3OG or C3OR confered significant protective effect against BL‐induced ROS production in HaCaT. *MAPK activity*: C3OG or C3OR significantly reduced BL‐induced phosphorylation of FAK, MAPKs, or IKKα.‐ *Inflammatory cytokines*: C3OG or C3OR significantly inhibited BL‐induced expression of TNF‐α, IL‐6, and IL‐8. *Apoptosis*: Both compounds decreased cleavage of FAK and PARP by limiting caspase‐3 activation (*No numeric values reported*)
Wu et al. (2024)	In vitro: Human skin keratinocytes (HaCaT cells) In vivo: 30 male Kunming mice (5 weeks old)	*Lycium barbarum polysaccharide (LBP)* In vitro*: 0.1, 1, and 10 mg/mL; 24 h pretreatment* In vivo*: Subcutaneous injection for 3 days at 50 mg/mL or 200 mg/mL*	BL: In vitro: 435–445 nm, 8000 lx, 11.2 W/cm^2^ In vivo: dorsal skin irradiated 3 days, 8 h/day, 10,000 lx	IG1—In vitro: HaCaT cells pretreated with LBP (0.1–10 mg/mL) for 24 h and then exposed to BL (435–445 nm; 8000 lx; 11.2 W/cm^2^). IG2—In vivo: Mice pretreated with subcutaneous LBP (50 or 200 mg/mL) for 3 days and then BL‐irradiated on dorsal skin	CG1‐In vitro controls: HaCaT untreated (CTR) HaCaT exposed to BL only HaCaT treated with LBP but not irradiated CG2‐In vivo controls: CTR (normal skin, no BL) BL (blue‐light only) BL + normal saline (vehicle control)	Cytotoxicity (MTT assay) In vitro outcomes: Cell morphology (microscopy, imaging) Apoptosis (Annexin V‐FITC kit) ROS production (DFCH‐DA assay) Antioxidant capacity (MDA and SOD levels) Autophagy (LC3B expression) (western blotting and immunofluorescence) Mitochondrial status (JC‐1) Mitophagy (mitochondrial and lisosomal colocalization, immunofluorescence) In vivo outcomes: Skin morphology (H&E and Masson) Antioxidant activity (SOD and MDA expression) Mitophagy (LC3B, PINK‐parkin pathway)	In vitro *experiments* *Cytotoxicity*: 1 mg/mL LBP pretreatment significantly ameliorates BL‐induced cytotoxicity. (*No numeric values reported*) In vitro findings: *Cell morphology*: LBP attenuated BL‐induced morphological damage. *Apoptosis*: LBP reduced the percentage of apoptotic cells compared with BL alone. *ROS*: LBP markedly suppressed BL‐induced ROS overproduction. *Antioxidant markers*: LBP prevented BL‐induced MDA increase and restored SOD activity. *Autophagy*: LBP reduced BL‐triggered autophagy (↓LC3B/LC3II). *Mitochondrial function*: LBP restored mitochondrial membrane potential and partially normalized BL‐induced intracellular Ca^2+^ elevation. *Mitophagy*: LBP attenuated excessive mitophagy induced by BL. (*No numeric values reported*) In vivo *experiment*: *Skin morphology*: BL caused epidermal and dermal damage; LBP (50–200 mg/mL) improved tissue architecture, with greater protection at 200 mg/mL *Collagen structure*: LBP prevented BL‐induced collagen fiber disorganization *Antioxidant activity*: LBP reversed BL‐mediated SOD inhibition and MDA elevation *Mitophagy*: LBP decreased BL‐induced LC3B expression and reduced activation of the PINK1–Parkin pathway. (*No numeric values reported*)

**TABLE 2:** Summary of clinical evidence on the photoprotective effects of natural compounds against visible light.

Study	Ingredient/Dose	Irradiation dose	Participants: *N* (mean age); sex	Fitzpatrick skin phototype (SPT); ethnicity	Intervention group (IG)	Comparison group (CG)	Outcome (measures)	Main results	Suitable for meta‐analysis
Kohli et al. (2017)	*Polypodium leucotomos* extract (PLE), 480 mg oral total (240 mg 2 h before +240 mg 1 h before irradiation)	VL (80–480 J/cm^2^); using a Fiber‐Lite Model 180 with a 150 W EKE lamp with filter GG400/3 mm and 3‐mm hot mirror	*N* = 22 healthy adults (NR); men and women included	I–III; (NR)	IG: Oral PLE (480 mg) before irradiation; UVB/UVA1/visible light applied to right side of the back on Day 3; assessments on Day 4 (Post‐PLE group)	CG: No PLE; identical UVB/UVA1/VL irradiation to left side of the back on Day 1; assessments on Day 2 (Pre‐PLE group)	*Pigmentation outcomes*: IGA of pigmentation Colorimetry (b*)	*Pigmentation*: VL did not induce pigmentation in SPT I–III. IGA pigmentation and colorimetry (b*) showed no detectable changes before or after PLE. No numerical VL data were reported, and no protective effect of PLE could be demonstrated under VL exposure^a^	No VL outcomes lack numerical data (no means or SD reported)
Mereniuk et al. (2018)	Topical Vitamin E cream (vitamin E 20.0% w/w; 114.0 mg α‐tocopherol equivalents/250 IU per gram)	VL incremental fluences: 55 J/cm^2^, 274 J/cm^2^, 549 J/cm^2^, and 1098 J/cm^2^ (irradiance 343 mW/cm^2^; 99.3% VL)	*N* = 10; (35.5); 63.6% female/36.4% male	IV (63.6%) and V (36.4%); Non‐hispanic (72.7%)	IG: Topical vitamin E cream applied at 2 mg/cm^2^ on one side of the back, 24 h and 30 min prior to VL exposure (1 cm^2^ test sites; randomized intra‐subject)	CG: Topical bland emollient (Eucerin cream, no vitamin E) applied at 2 mg/cm^2^ on the contralateral side, 24 h and 30 min before VL exposure (1 cm^2^ test sites)	*Pigmentation outcomes*: Visual assessment of lowest fluence inducing pigmentation (Day 7) Colorimetry (ΔL*) at 549 J/cm^2^ Colorimetry (ΔL*) at 1098 J/cm^2^	*Pigmentation*: No significant differences between IG and CG for lowest fluence inducing pigmentation (IG: 480 J/cm^2^ (SD not reported), CG: 370 J/cm^2^ (SD not reported) *p* = 0.182). No differences in pigmentation intensity at 549 J/cm^2^ (IG: 3.36; CG: 3.73; *p* = 0.778) or 1098 J/cm^2^ (IG: 6.85; CG: 6.23; *p* = 0.585) No differences in ΔL* pigmentation intensity at 1098 J/cm^2^: IG: 6.85 (SD not reported), CG: 6.23 (SD not reported) (*p* = 0.585) Vitamin E did not reduce VL–induced pigmentation	No Outcomes reported without SD, only means and *p*‐values
Campiche et al. (2020)	2 active formulations: Microalgae extract formulation: Base formulation +3% *Scenedesmus rubescens* dry extract (microalgae extract) (2 mg/cm^2^ daily) Niacinamide formulation: Base formulation +3% niacinamide (2 mg/cm^2^ daily)	BL irradiation: 60 J/cm^2^ per day × 4 days (total 240 J/cm^2^), 450 nm LED, irradiance 54 W/m^2^ at 9 cm	33 healthy subjects; (21–41 years); 100% female	III (54.5%) and IV (45.5%) 18 caucasians, 6 asians, 9 mixed	Two active topical formulations applied on designated forearm zones: IG1: Microalgae extract formulation IG2: Niacinamide formulation Both formulations were applied daily at 2 mg/cm^2^ during the conditioning phase (Day‐6 to Day‐1) and recovery phase (Day 4 to Day 28); not applied during irradiation days (Day 0 to Day 3) Active formulations were randomly assigned to inner forearm areas in a double‐blind, intra‐subject randomized design	CG: Base cream without active ingredients applied daily at 2 mg/cm^2^ except irradiation days Assigned to a contralateral forearm zone in the same double‐blind, intra‐subject randomized design	*Erythema outcomes*: Chromameter a* (redness) Hyperspectral imaging: haemoglobin content, oxygen saturation *Pigmentation outcomes*: Hyperspectral imaging of melanin content Chromameter L*, a*, b* ITA° day 3 ΔITA° (change from baseline) Day 3 ΔE (overall colour change) Clinical photographic assessment	*Erythema*: BL increased redness (a*) in all groups (*p* < 0.001). IG2 reduced a* versus CG (*p* < 0.05); IG1 showed a trend (*p* = 0.079). Haemoglobin increased post‐irradiation in all groups; IG2 showed a borderline lower increase (*p* = 0.096). No numeric erythema values were reported. *Pigmentation*: Melanin content increased in all groups with no significant differences. L* decreased (darkening) in all arms; active groups showed slightly less reduction (no values reported). a* increased; niacinamide reduced a* versus CG (*p* < 0.05). b* showed no relevant group differences. ΔITA° (Day 3): IG1 = −11.86; IG2 = −13.96; CG = −16.79 (SD not reported); actives showed smaller ITA° decreases Δ*E* was significantly lower in IG1 versus CG (*p* < 0.05*) though no numerical values were provided. Photographs showed visibly reduced darkening in IG1 and IG2 versus CG	No — Numeric data only reported for ΔITA° (no SD); all other outcomes lack quantitative values
Lyons et al. (2022)	Topical antoxidants blend (AO, diethylhexyl syringylidene malonate, vitamin E, ascorbyl palmitate) at 0.5%, 1% and 2%	VL + UVA1 irradiation: 480 J/cm^2^ for SPT I–III; 320 J/cm^2^ for SPT IV–VI; irradiance ~86 mW/cm^2^; spectral composition 97.3% VL, 2.0% UVA1, 0.7% IR	20 healthy subjects (NR); 40% female/60% male	I‐III (50%); IV–VI (50%) NR	IG1: AO blend at 0.5% IG2: AO blend at 1% IG3: AO blend at 2% Applied to separate marked sites on the back; occluded 1 h before exposure; assessments at immediate, 24 h, and Day 7	CG: untreated site and placebo formulation site, treated identically but without active AO Biopsied control site for histology comparison with 2% AO	*Erythema outcomes*: IGA Colorimetry (Δa) Diffuse reflectance spectroscopy (DRS) AUC (erythema) *Pigmentation outcomes*: IGA pigmentation Colorimetry (ΔITA) DRS AUC (pigment/darkness) *Histology/Molecular outcomes* COX‐2 staining MART‐1 melanocyte count Cyclin D1 staining (biopsy at 24 h from 2% AO versus placebo)	*In SPT I–III*: IGA showed trend (ns). 2% AO significantly reduced erythema by Δa and DRS AUC immediately post‐irradiation (*p* < 0.0167); IGA erythema showed a non‐significant trend. Pigmentation changes were minimal *In SPT IV–VI*: 2% AO significantly reduced immediate pigment darkening (ΔITA *p* < 0.0167) and showed reduction in delayed tanning at Day 7 (ΔITA *p* = 0.07) *Histology*: no significant differences in COX‐2 or MART‐1; Cyclin D1 reduction approached significance only in SPT IV–VI (*p* = 0.06). AO blends mitigated erythema (SPT I–III) and pigmentation (SPT IV–VI).	No No numerical data (means/SD) reported for any erythema, pigmentation, or histology outcomes; multiple interventions; split phototype cohorts
Ruvolo et al. (2022)	*Product A*—Control sunscreen (no antioxidants): SPF 50 chemical sunscreen base. *Product B*—3‐AO sunscreen: SPF 50 + antioxidant blend: 1% diethylhexyl syringylidene malonate; 0.25% vitamin E; 0.01% ascorbyl palmitate *Product C*—5‐AO sunscreen: SPF 50 + enhanced antioxidant blend: 0.5% diethylhexyl syringylidene malonate; 0.25% vitamin E; 0.01% vitamin C; 0.025% licochalcone A; 0.01% glycyrrhetinic acid *Product D*—Tinted sunscreen: SPF 20 with TiO_2_ and iron oxides	380 J/cm^2^ total dose; irradiance ~95 mW/cm^2^; spectral composition 96.3% VL (400–700 nm), 1.4% UVA1 (340–400 nm), and 2.28% IR (700–1800 nm) using a LS1000 solar simulator	*N* = 10, 9 completed (NR); 100% female	IV (33.3%) V (33.3%) VI (33.3%) NR	IG1: SPF 50 + 3‐AO blend IG2: SPF 50 + 5‐AO blend IG3: Tinted sunscreen with TiO_2_ and iron oxides All applied occlusively for 24 h, then re‐applied at 2 mg/cm^2^ prior to irradiation	CG1: untreated irradiated skin (no product) CG2: SPF 50 sunscreen without antioxidants (vehicle control)	*Erythema outcomes*: DRS Δ oxy‐haemoglobin DRS AUC *Pigmentation outcomes*: (IGA) pigmentation (0–8 scale) DRS (Δ melanin content) DRS AUC (relative dyschromia/darkness)	*Erythema*: IG2 (5‐AO) and IG3 (tinted) showed reduced erythema versus untreated and no‐AO controls CG (*p* ≈ 0.02–0.07) IG2 and IG3 showed lower erythema AUC versus controls (*p* < 0.05 at several time points) *Pigmentation*: IG3 (tinted) showed significantly lower pigmentation than untreated skin (*p* < 0.05 at all time points) IG2 (5‐AO) showed comparable or better protection than IG3 (*p* < 0.05 where stated) IG2 had significantly lower Δ melanin at Day 7 compared with no‐AO sunscreen (*p* < 0.05). IG2 provided the largest reduction in dyschromia, outperforming IG3 and controls (*p* < 0.05) IG2 (5‐AO) offered the strongest and most consistent protection against VL + UVA1–induced erythema and pigmentation (multiple outcomes *p* < 0.05).	No Means and SD not provided; figures lack numerical axis values; quantitative extraction not possible even via digitization
Ezekwe et al. (2024)	*Product A*: SPF60 untinted sunscreen + tocopherol and *Cassia alata* leaf extract. No iron oxides. *Product B*: SPF50 tinted sunscreen + zinc oxide 12% + tocopherol and *Cassia alata* extract +4% iron oxides *Product C*: SPF50 untinted sunscreen + zinc oxide 12% + tocopherol and *Cassia alata* extract. No iron oxides *Product D*: SPF50 tinted sunscreen +11% titanium dioxide + tocopherol and *Cassia alata* extract +1% iron oxides	320 J/cm^2^ total dose; irradiance ~80 mW/cm^2^; spectral composition 95.5% VL (400–700 nm), 3.5% UVA1 (370–400 nm), and 1.0% IR (700–1600 nm) using a LS1000 xenon arc solar simulator	12 healthy subjects (32.5 years); 83% female/17% male	III (67%) and IV (33%) NR	IG‐A: Product A (SPF 60, untinted, no iron oxides) IG‐B: Product B (SPF 50, tinted, 4% iron oxides) IG‐C: Product C (SPF 50, untinted, no iron oxides) IG‐D: Product D (SPF 50, tinted, 1% iron oxides) All products applied at 2 mg/cm^2^, left for 30 min before irradiation. Duplicate areas for A, B, and untreated control allowed biopsy.	CG: Untreated irradiated skin (control site)	*Erythema outcomes*: IGA erythema score (0–5 scale) Colorimetry: Δa* DRS: Δ oxy‐hemoglobin DRS: relative erythema AUC (400–700 nm) *Pigmentation outcomes*: IGA pigmentation (0–5 scale) Colorimetry: ΔITA DRS: relative dyschromia AUC (400–700 nm) DRS: immediate pigment darkening (IPD) visualized on Day 0 *Histology/Molecular outcomes* COX‐2 staining MIB1 (cell count) Sox10 (melanocyte count) Fontana‐Masson (melanin pigment intensity)	*Erythema*: IGA erythema: CG = 2.2; lower in IG‐C (1.4) and IG‐D (1.0) (*p* < 0.05) Δa*: CG = 2.4; lowest in IG‐D (0.7) and IG‐C (1.3) (*p* < 0.05) Erythema was highest in the CG (1.05). Products C (0.60) and D (0.55) showed the greatest erythema reduction versus control (*p* < 0.05) CG showed the highest AUC (55). All products reduced AUC, especially D (47) and C (48) versus control (55) *Pigmentation*: IGA scores: At Day 0, IG‐D (1.0) < CG (2.2) (*p* < 0.05). At Day 7, all IGs<CG (3.1). At Day 14, only IG‐D (0.9) remained significantly lower than CG (1.9) ΔITA: Day 0 pigmentation lowest in IG‐D (−4) versus CG (−12) (*p* < 0.05) DRS: At Day 7, IG‐D [12] and IG‐B [14] < CG [24] (*p* < 0.05). IG‐D showed the least darkening; CG showed the highest *Histology*: MIB1: IG‐A showed higher proliferation versus CG (*p* < 0.05) COX‐2, Sox10, Fontana‐Masson: No significant differences versus CG IG‐D (tinted, 1% iron oxides) provided the most consistent protection against VL + UVA1–induced erythema and pigmentation	No Numeric means were extracted visually from figures, but SD/SEM values are not provided. Insufficient quantitative data for meta‐analysis

*Note:* Results from erythema (MED) and pigmentation (colorimetry) refer exclusively to UVB irradiation. VL and UVA1 light doses applied may not have been sufficient to induce erythema and pigmentation responses in individuals with phototypes I–III. Table only analyzes VL results.

### 3.2 Participants' Characteristics

Overall, the clinical evidence synthesized in this review comprised a total of 107 participants across six interventional phototesting studies. Age data were explicitly provided for 51.4% of the total population (55/107) across three of the six included studies; these reported mean ages of 32.5 and 35.5 years and a specific inclusion range of 21 to 41 years. Sex distribution was reported in five studies, accounting for 85 participants, of whom approximately 79% were female, reflecting a predominance of women in VL photoprotection trials.

Regarding skin phototype, all studies included participants with all Fitzpatrick skin phototypes, although most trials focused on intermediate to darker phototypes. Phototypes I–III were primarily represented in studies evaluating erythema and early pigmentary responses, whereas phototypes III–VI were more frequently included in studies assessing visible light–induced pigmentation and dyschromia. Several trials specifically stratified outcomes by phototype group, highlighting differential erythema responses in lighter phototypes and more pronounced pigmentation effects in darker phototypes. Ethnicity was inconsistently reported and therefore could not be quantitatively summarized.

### 3.3 Risk of Bias Assessment

**FIGURE 2:**
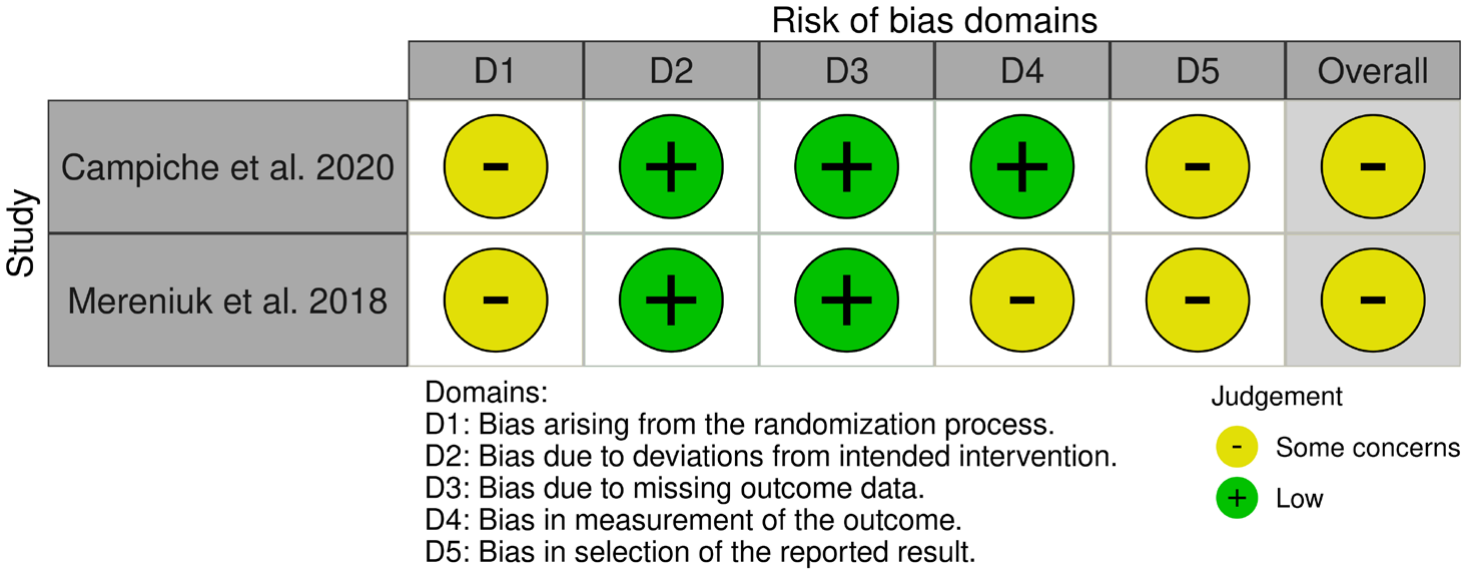
ROB‐2 traffic light plot for randomized controlled trials.

Risk of bias was assessed using the ROB‐2 tool for the two randomized controlled trials included [18, 19] (Figure 2). Both studies were judged to raise some concerns, mainly due to insufficient reporting of the randomization process and lack of protocol preregistration. The reviewers achieved a 91.6% agreement in the pair‐wised evaluation.

**FIGURE 3:**
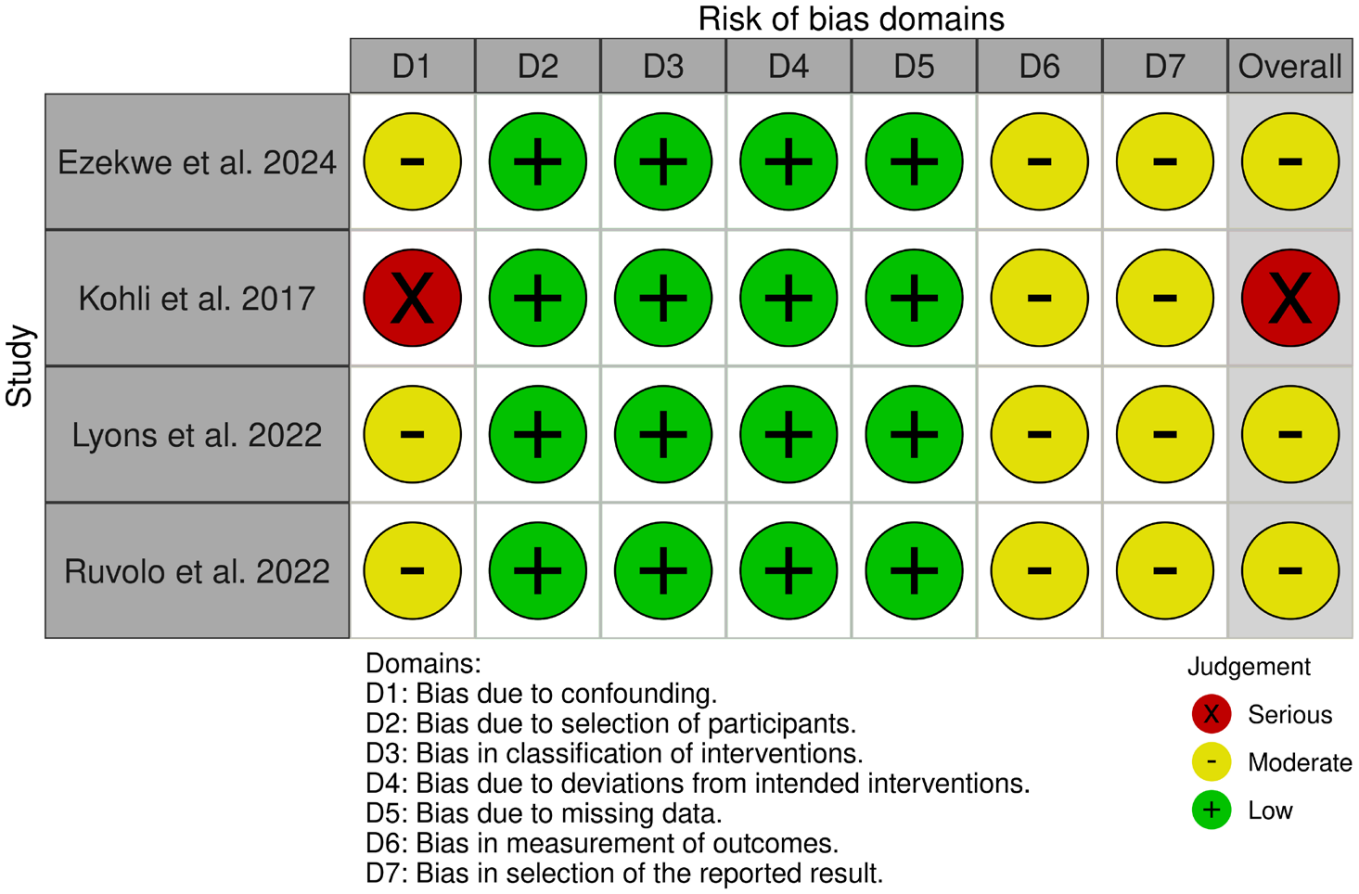
ROBINS‐I traffic light plot for non‐randomized controlled trials.

Risk of bias in non‐randomized studies was assessed using the ROBINS‐I tool (Figure 3). Three studies were judged to be at moderate risk of bias [14, 20, 21], mainly due to potential residual confounding from non‐randomized allocation of treatment sites, partial reliance on subjective clinical outcomes, and lack of protocol preregistration. One study showed a serious risk of bias because of its fixed pre–post design without a concurrent control group, which introduces substantial temporal confounding [22]. The percentage of agreement between reviewers was 92.25%. A global overview of these methodological judgments is visually consolidated in Figure 2 (ROB‐2 summary) and Figure 3 (ROBINS‐I traffic light plot). As illustrated in this Risk of Bias assessment, the “serious risk” and “low‐to‐moderate certainty” designations within the natural VL photoprotection literature are largely driven by pioneering but methodologically limited studies, such as the pre–post design in Kohli et al. [22]. By isolating these study‐specific flaws in the text, we ensure they do not falsely penalize the well‐established clinical grade (Level 1) of the therapeutic agent as a global entity.

The OHAT assessment showed that most preclinical studies had a probably high risk of bias, mainly due to the lack of randomization and blinding and the absence of protocol preregistration, leading to potential selective reporting. In contrast, exposure characterization was consistently judged as low risk of bias, and outcome assessment relied largely on standardized, objective laboratory methods (File S3). The percentage of agreement was 95.45%.

### 3.4 Certainty of Evidence (GRADE) for Clinical Outcomes

For VL–induced pigmentation outcomes [14, 18, 19, 20, 21, 22], the certainty of evidence was rated as moderate. The certainty was downgraded due to methodological limitations, as most studies presented moderate risk of bias and one key study showed serious risk due to confounding, as well as serious imprecision related to small sample sizes and incomplete reporting of variability measures. No downgrading was applied for inconsistency or indirectness, as all studies reported concordant effects using clinically relevant outcomes.

For VL–induced erythema outcomes [14, 18, 20, 22], the certainty of evidence was rated as low. Although all studies consistently showed attenuation of erythematous responses following treatment with antioxidant‐based formulations or tinted products, the evidence was downgraded due to methodological limitations, including moderate to serious risk of bias and serious imprecision related to small sample sizes and incomplete reporting of variability measures. No downgrading was applied for inconsistency or indirectness.

For molecular biomarkers of VL–induced skin damage [22], the certainty of evidence was rated as very low, due to reliance on a single small non‐randomized study with serious risk of bias and very serious imprecision related to limited sample size and incomplete reporting of variability measures. Although a protective effect was observed, confidence in the magnitude and reproducibility of this finding remains very limited.

For all outcomes the percentage of agreement between reviewers was 100%. File S2 shows the detail for the GRADE findings of each outcome.

### 3.5 Results by Natural Agent

The following results synthesize the clinical and preclinical evidence regarding the efficacy of natural agents in mitigating cutaneous damage induced by VL and HEVL. The analysis focuses on key photobiological variables, including pigmentation, erythematous response, and relevant molecular biomarkers, integrating mechanistic insights and statistical data where available (Tables 1 and 2).

#### 3.5.1 Polyphenolic Compounds

Polyphenols represent the most robustly documented class of natural agents in this review, supported by a total of 16 studies (13 preclinical and 3 clinical).

Preclinically, these compounds have demonstrated a multi‐targeted capacity to protect cellular viability and prevent VL‐induced oxidative stress, neutralizing ROS and activating endogenous antioxidant defenses through various molecular pathways. This evidence is predominantly led by *Polypodium leucotomos* extract (PLE, Fernblock^(R)^), which has demonstrated significant cytoprotective effects when administered as a preventive treatment against VL‐induced damage. In human fibroblasts, this extract preserves cell morphology and viability while inhibiting VL‐induced alterations in the expression of critical extracellular matrix (ECM) proteins, including matrix metalloproteinase‐1 (MMP‐1), cathepsin K (CTSK), fibrillin‐1/2 (FBN1/2), and elastin (ELN) [23]. Moreover, when human fibroblasts and melanocytes were exposed to HEVL, Fernblock^(R)^ preserved their viability, reducing ROS production and p38 MAPK phosphorylation, thereby mitigating oxidative stress [24]. Similarly, hydroxytyrosol, a polyphenol sourced from the *Olea europaea* fruits, and a constituent of olive oil, has been shown to protect the viability of keratinocytes and fibroblasts, effectively downregulating ECM markers such as MMP‐1, MMP‐12, collagen type I and the cell proliferation marker PCNA [25]. Flavonoid components, including apigenin, chrysin, and galangin, demonstrated potent ROS‐scavenging properties; specifically, chrysin and apigenin preserved viability of human melanocytes, efficiently suppressing singlet oxygen overproduction induced by VL [26]. Furthermore, pre‐treatment of human BL‐exposed keratinocytes with a *Helichrysum arenarium* extract (rich in apigenin and galangin) increased cell survival rate, attenuated intracellular calcium flux by antagonizing TRPV1‐mediated signaling, significantly reduced ROS production and counteracted MAPK pathway alterations [27]. Another flavonoid with antioxidant, anti‐angiogenic, and antitumor potential effects is licochalcone A (LicA), isolated from the roots of *Glycyrrhiza inflate* [28]. Mann et al. [29] found that treating human dermal fibroblasts with a licorice extract solution rich in LicA prior to VL exposure decreased ROS production and markedly induced Nrf2 activation. Similarly, the *Melissa officinalis* phytocomplex, rich in rosmarinic acid, prevented ROS overproduction and inhibited the compensatory overexpression of Nrf2 in keratinocytes irradiated with BL, suggesting a high direct scavenging efficiency that preserves cellular redox balance [30]. Another example is Edafence^(R)^ (EDA), a specialized flavonoid‐rich phytocomplex obtained from *Deschampsia antarctica* that has been shown to maintain cell viability, preserving mitochondrial morphology and preventing mitochondrial membrane hyperpolarization in both BL‐exposed human fibroblasts and melanocytes [31]. Furthermore, *Caesalpinia sappan* extract (CSE), of which the primary constituent is brazilin, and *Curcuma longa* L. extract, rich in curcuminoids, a group of polyphenols,^(R)^ enhance cellular antioxidant capacity by increasing superoxide dismutase (SOD) activity and DPPH radical scavenging power in human fibroblasts, thereby improving their viability and reducing BL‐induced ROS overproduction [32, 33].

Natural polyphenolic compounds contribute to genomic stability and exhibit anti‐inflammatory properties. Anthocyanins (cyanidin derivatives), isolated from the fruits of *Prunus serrulata* L. var. *tomentella* Nakai, downregulated the expression of markers such as MAPKs, FAK, and IKKα, inhibited the production of pro‐inflammatory cytokines (TNF‐α, IL‐6, and IL‐8), and suppressed cellular apoptosis via caspase‐3 activation Lee et al. [34]. This efficacy underscores the potent ability of polyphenolic compounds to attenuate VL‐induced intracellular signaling, most notably the MAPK signaling axis, which consequently limits downstream oxidative DNA damage and maintains cellular homeostasis. Additionally, the above‐mentioned compound hydroxytyrosol also prevented oxidative DNA damage, reducing 8‐Hydroxy‐2′‐Deoxiguanosine (8‐OHdG) in a dose‐dependent manner [25].

In terms of pigmentary dynamics, scientific evidence highlights the capacity of natural compounds to intercept signaling initiated by specific photoreceptors, such as OPN3, thereby modulating the melanogenic cascade from its incipient stages of photochemiexcitation. In melanocytes exposed to BL, Fernblock^(R)^ significantly restored basal expression of the opsin‐3 photoreceptor, attenuated melanin overproduction, and provided protection against DHICA‐melanin oxidation [24]. Similarly, both chrysin and apigenin protected human melanocytes from melanin photosensitization‐induced damage and membranes photooxidative damage triggered by VL [26]. Edafence^(R)^ also prevented BL‐induced hyperpigmentation through reduction of BL‐induced darkening of extra and intracellular melanin pigments and reduction of BL‐induced p38 melanogenic pathway activation [31]. Furthermore, Pycnogenol, derived from *Pinus pinaster* bark, exerted an inhibitory effect on pigmentation by reducing tyrosinase activity and suppressing key mediators such as endothelin‐1 (ED1) and peroxisome proliferator‐activated receptor (PPAR) isoforms [35].

These findings are clinically translated through *Polypodium leucotomos* extract (PLE, Fernblock^(R)^). In randomized trials, oral administration of 480 mg PLE significantly reduced the mean IGA erythema score and colorimetric erythema intensity [22]. Furthermore, PLE demonstrated a profound suppression of molecular damage, including a marked reduction in CPD, sunburn cells, and in proliferative markers such as PCNA and Ki67 [22]. Regarding pigmentary variables in dark skin phototypes (SPT) IV–VI, Mohammad et al. [36] confirmed that PLE significantly attenuated PPD and significantly reduced melanin intensity. Nevertheless, Kohli et al. [22] noted that in SPT I–III, VL failed to induce macroscopic pigmentation, suggesting that in lighter skin, the efficacy of polyphenols is primarily targeted at sub‐clinical DNA protection and anti‐inflammatory pathways.

#### 3.5.2 Polysaccharides and Amino‐Acid Derivatives

Beyond polyphenolic interventions, the preservation of cellular integrity and genomic stability has emerged as a primary therapeutic target for polysaccharides and amino acid derivatives. Preclinical research on a polysaccharide (LBP) extracted from the plant *Lycium barbarum* demonstrated that pretreatment of keratinocytes with this compound not only protected their viability from BL‐induced damage but also modulated intracellular activity of superoxide dismutase (SOD) and malondialdehyde (MDA), promoting a reduction in ROS production, improved mitochondrial status and attenuated BL‐induced mitophagy and autophagy increased levels. Consistent with these in vitro results, in vivo research with LBP confirmed its photoprotective efficacy against BL: it preserved mouse skin morphology, prevented collagen fibers disarrangement induced by BL, prevented alterations on antioxidant enzymes (SOD and MDA) and inhibited mitophagy through reduction of LC3B and PINK1‐parkin pathway [37].

Regarding amino acid derivatives, in vitro incubation of human keratinocytes with ectoine demonstrated substantial protection against VL‐induced DNA damage. Ectoine functions as a molecular chaperone to stabilize the hydration shell of cellular macromolecules, thereby safeguarding the proteome and genome against radiation‐induced osmotic and genotoxic stress [38]. Similarly, L‐ergothioneine, a sulfur‐containing derivative of L‐histidine, has exhibited significant dose‐dependent DNA protection against VL‐induced genotoxicity [38]. Collectively, these osmoprotectants provide a biophysical shield that complements the biochemical neutralization provided by traditional antioxidants.

#### 3.5.3 Vitamins and Carotenoids

Current evidence regarding vitamin‐based interventions underscores a significant discrepancy between monotherapy and synergistic multi‐component formulations. Specifically, topical application of α‐tocopherol (vitamin E) was found to be ineffective as a monotherapy, showing no clinical impact on the minimal pigmentary dose (MPD) or colorimetric darkening intensity relative to the vehicle [19]. In contrast, multi‐component formulations containing niacinamide demonstrated a superior capacity to modulate vascular variables, significantly attenuating BL‐induced erythema and mitigating the decline in individual typology angle (ITA°) values following repetitive exposure to HEVL radiation [18].

The clinical advantage of complex systems is further corroborated by the efficacy of penta‐antioxidant enriched sunscreens, incorporating vitamins C and E alongside LicA, which achieved a reduction in relative dyschromia and melanin deposition compared to non‐antioxidant controls [21]. Regarding carotenoids, β‐carotene has been identified as a potent singlet oxygen quencher, enhancing melanocyte survival and suppressing oxidative stress mediated by melanin photosensitization under VL irradiation [26]. These data suggest that while individual vitamins may lack sufficient potency to counteract the high‐energy visible spectrum, their integration into multi‐active antioxidant networks provides a robust defense against both immediate vascular responses and delayed pigmentary alterations.

#### 3.5.4 Antioxidant Blends, Algal Extracts and Others

The development of multi‐component antioxidant networks and the utilization of bio‐marine extracts signify a shift toward a more comprehensive photoprotective paradigm that extends beyond traditional filters.

Initial in vitro assessments have established the superior efficacy of synergistic blends in neutralizing radiation‐induced stress. Specifically, a combination of feverfew ( *Tanacetum parthenium* ), soy ( *Glycine soja* ), and gamma‐tocopherol, alone or included into a UV sunscreen, demonstrated a potent capacity to reduce both ROS generation and the release of pro‐inflammatory markers such as IL‐1α and MMP‐1 in human epidermal equivalents exposed to VL, significantly outperforming baseline UV filters [39]. Complementing these findings, Pu'er tea extract, with its complex polyphenolic and flavonoid profile, has demonstrated high DPPH radical scavenging activity and strong reducing power against BL, making it an excellent candidate for inclusion in photoprotective formulations [33].

Preclinical research has also highlighted the capacity of mannitol, a sugar alcohol or polyol, to protect human keratinocytes from damage induced by UVA/VL and VL spectral ranges [38].

These mechanistic observations are further substantiated by clinical evaluations focusing on macroscopic skin parameters. Topical application of a multi‐active antioxidant blend incorporating LicA demonstrated significant efficacy in mitigating erythema (Δa*) and stabilizing the individual typology angle (ΔITA) [14]. Notably, these clinical outcomes revealed a critical dose–response relationship, where superior protection over placebo was exclusively achieved at higher concentrations, highlighting the necessity of optimized formulation potencies [14]. In parallel, the inclusion of *Scenedesmus rubescens* microalgal extract has been clinically proven to mitigate ITA° decline and reduce overall color change (Δ*E*), reinforcing the structural protection required to maintain skin homeostasis following repetitive high‐energy exposure [14, 18].

#### 3.5.5 Synergy With Physical Blockers: The Iron Oxide Variable

The inclusion of physical mineral blockers proved to be a critical variable in providing immediate and sustained clinical protection against the VL spectrum. Clinical evaluations demonstrated that a tinted formulation containing 1% iron oxides significantly reduced colorimetric erythema (Δa*) and yielded significantly less hyperpigmentation across all evaluated time points [20].

These clinical results are supported by in vitro studies demonstrating that tinted sunscreens achieved up to 100% protection against VL‐induced DNA damage, whereas non‐tinted formulations containing only UV filters conferred significantly lower levels of protection [38]. Despite the barrier efficiency of minerals, spectroscopic analysis revealed that organic sunscreens enriched with a penta‐antioxidant system achieved a reduction in melanin content at Day 7 that was statistically non‐inferior to tinted mineral benchmarks, suggesting that potent biological antioxidants can effectively manage the oxidative cascade that penetrates physical barriers [21].

## 4 Discussion

**FIGURE 4:**
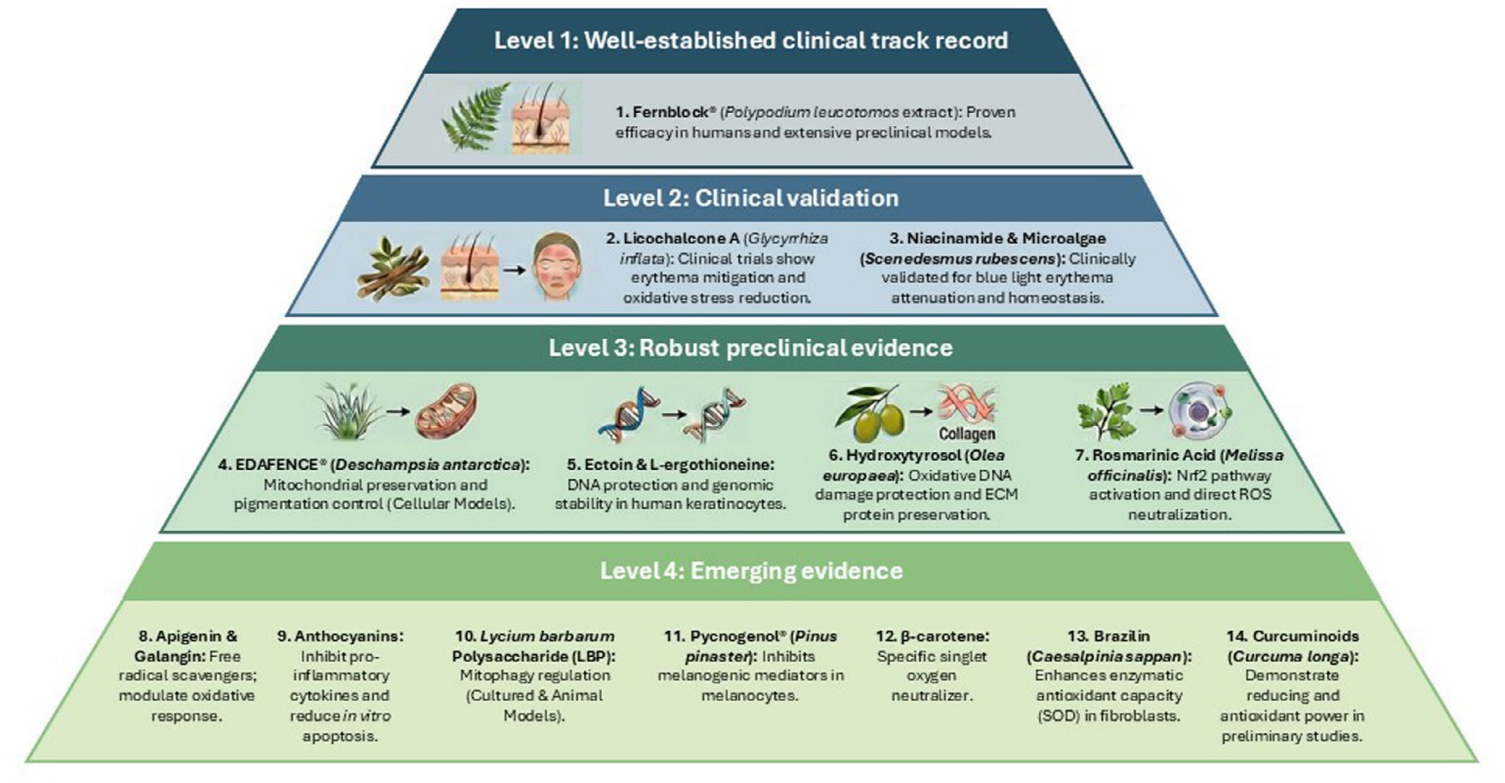
Pyramid of evidence of the natural ingredients included in this systematic review, classified according to the efficacy and certainty of the evidence demonstrated. Natural compounds were organized into four progressive levels: Level 1: Well‐established clinical track record (with data limitations), Level 2 (Clinical validation), Level 3 (Robust preclinical evidence), and Level 4 (Emerging evidence). For each compound, the predominant experimental model and the principal mechanisms of action supporting its photoprotective effect are indicated. Level 1 reflects extensive clinical usage and consensus alongside robust mechanistic data, despite current clinical trial methodological limitations (e.g., risk of bias or small sample sizes). Key: ECM, extracellular matrix; Nrf2, nuclear factor erythroid 2‐related factor 2; SOD, superoxide dismutase.

The present systematic review highlights polyphenolic compounds, specifically PLE (Fernblock^(R)^) as the class of natural agents with the most comprehensive and consistent scientific support for VL photoprotection. This hierarchy of evidence is substantiated by a robust translational framework linking mechanistic preclinical findings, such as the inhibition of MMP‐1, regulation of the BL receptor OPN3, and preservation of mitochondrial membrane potential, to meaningful clinical outcomes, including the reduction of erythema and the suppression of genotoxic biomarkers like CPDs. However, it is critical to differentiate between the robust molecular consensus of PLE and the specific methodological constraints of its available VL clinical trial. The serious risk of bias identified under our ROBINS‐I framework (due to a fixed pre‐post design without a concurrent control) and the statistical imprecision (yielding Moderate/Low certainty in GRADE due to *N* = 22) are strictly confined to the isolated trial by Kohli et al. [22]. These limitations reflect the historical lack of standardized protocols for visible light phototesting at the time the study was conducted, rather than a deficiency in the therapeutic potential of the extract itself. Therefore, while explicitly acknowledging the methodological limitations of the available clinical data, PLE is categorized under Level 1: Well‐established clinical track record with data limitations (Figure 4). This position is supported by its extensive clinical consensus and robust mechanistic translation in dermatological care, rather than high‐certainty clinical trial evidence alone.

A critical analysis of the evidence underscores the “failure of antioxidant monotherapy,” exemplified by isolated Vitamin E, which failed to demonstrate significant protection against VL‐induced pigmentation. This biological limitation is attributable to the complex, multifaceted pathophysiology of skin damage in this spectrum. The simultaneous activation of diverse oxidative and inflammatory pathways likely supersedes the quenching capacity of any single molecule. Conversely, multicomponent antioxidant blends demonstrate superior efficacy by eliciting synergistic effects across diverse cellular targets, such as the p38 MAPK signaling axis and Nrf2‐mediated redox homeostasis. This synergy is further optimized by integrating biological actives with physical blockers like iron oxides, establishing a dual‐spectrum defense model where the external barrier minimizes photon absorption while natural agents neutralize the residual oxidative cascade.

Despite the consistency of the observed benefits, the primary technical limitation of this review is the inability to perform a quantitative meta‐analysis. This obstacle stems from a systemic lack of standardization in numerical data reporting within clinical literature; specifically, the frequent omission of standard deviations (SD) or standard errors (SEM) is frequent, alongside the reliance on subjective visual scales and heterogeneous irradiation sources, preclude rigorous statistical pooling. Furthermore, substantial heterogeneity exists regarding irradiation sources, characterized by varying spectral power distributions and the frequent inclusion of residual UVA1 wavelengths, alongside irreproducible irradiance levels (mW/cm^2^) and disparate cumulative doses (J/cm^2^). Outcome assessments also lack harmonization, drifting between subjective visual clinical scales (IGA) and quantitative colorimetry (ΔITA, Δa*). These baseline differences in dosing regimens, formulation compositions, and experimental protocols pose severe challenges to cross‐study comparability and the direct translation of findings into clinical practice. Addressing this widespread lack of methodological and reporting standardization is vital for advancing the field towards reproducible, synthesis‐ready data. Furthermore, clinical cohorts exhibit a stark female overrepresentation (83%), yet they largely fail to account for the hormonal milieu (e.g., oral contraceptives, menstrual cycle phase, or menopause). Given that estrogen and progesterone significantly prime melanocytes toward hyper‐reactivity, the lack of hormonal stratification represents a major confounding variable in pigmentation kinetics. Thus, the impact of chronological age remains critically understudied. The age‐dependent decline in endogenous antioxidant defenses and DNA repair capacity likely lowers the threshold for VL‐induced inflammaging, yet the majority of trials utilize healthy, middle‐aged volunteers, potentially underestimating the damage in older populations.

Additionally, regarding photoprotection strategies, limitations of this review include the fact that most of the included studies were conducted using short‐term assessments under controlled experimental conditions, which may not accurately represent real‐world exposure scenarios. Furthermore, the absence of standardized models for VL irradiation and the variability in dosing regimens, formulation compositions, and irradiation protocols poses challenges to cross‐study comparability and the translation of findings into clinical practice. To overcome these data fragmentations, address the confounding variables identified in current literature, and pave the way for future quantitative meta‐analyses, upcoming clinical trials evaluating natural agents against VL must adopt more rigorous, standardized, and real‐world reproducible frameworks. Future research protocols should mandate the precise characterization of physical parameters, explicitly disclosing the full spectral power distribution to rule out ultraviolet contamination, along with exact irradiance (mW/cm^2^) and cumulative doses (J/cm^2^) across extended timelines. To eliminate the substantial temporal confounding and selection bias found in early literature, the implementation of randomized controlled designs with concurrent control groups is essential. Furthermore, data reporting standards must transition away from subjective visual clinical grading toward objective, automated metrics, ensuring the mandatory disclosure of mean values and standard deviations (SD) for all colorimetric (ΔITA, Δa*) and molecular biomarkers. Methodological designs must also evolve to evaluate synergistic multicomponent formulations over long‐term, real‐world exposure scenarios, incorporating strict cohort stratification that accounts for age‐dependent repair kinetics and the hormonal milieu (such as menstrual cycle phases, oral contraceptives, or menopausal status). Adherence to these standardized reporting and experimental requirements is vital to establish true cross‐study comparability, minimize statistical imprecision, and ultimately facilitate the robust synthesis of clinical datasets into definitive, evidence‐based dermatological guidelines.

## 5 Conclusions

In conclusion, while current evidence strongly supports the therapeutic integration of antioxidant blends and polyphenols such as PLE (Fernblock^(R)^), the field must transition toward personalized photoprotection. Future research must adopt standardized reporting protocols and inclusive clinical designs that account for sex‐based biological differences, age, and hormonal status. Such rigor is essential to consolidate global therapeutic protocols for the management of VL‐induced dermatoses and photoaging.

## Author Contributions

Conceptualization: A.R.‐L., A.Z. and S.G.; Data curation: A.R.‐L. and A.Z.; Formal analysis: A.R.‐L. and C.G.‐M.; Methodology: J.‐C.H.‐R. and C.G.‐M.; Software: J.‐C.H.‐R. and C.G.‐M.; Supervision: A.R.‐L. and S.G.; Validation: A.R.‐L., H.W.L. and S.G.; Visualization: J.‐C.H.‐R. and C.G.‐M.; Writing – original draft: A.R.‐L. and A.Z.; Writing – review and editing: A.R.‐L., A.Z., J.‐C.H.‐R., C.G.‐M., H.W.L. and S.G.

## Conflicts of Interest

H.W.L. has served as an investigator for Incyte, La Roche Posay, Pfizer, and PCORI; as a consultant for ISDIN, Beiersdorf, Ferndale, L'Oréal, Eli Lilly, Zerigo Health, Skinosive, Kenvue, Cantabria Labs, NAOS, and Boehringer Ingelheim; and as a speaker on general educational session for La Roche‐Posay, Cantabria labs, Pierre Fabre, NAOS, Uriage, Pfizer, ISDIN, Clinuvel. S.G. serves as a consultant for Cantabria Labs.

## Supporting information


**File S1:** phpp70113‐sup‐0001‐SupinfoS1‐S3‐TableS1‐S2‐FigureS1.docx.
**Table S1:** Detailed literature search strategy.
**Table S2:** Certainty of evidence (GRADE) for clinical outcomes.
**Figure S1:** OHAT traffic light plot for preclinical studies.

**Data S1:** phpp70113‐sup‐0002‐supinfo.pdf.

## Data Availability

The data that supports the findings of this study are available in the Supporting Information of this article.

## References

[1] E. Austin , A. N. Geisler , J. Nguyen , et al., “Visible light. Part I: Properties and Cutaneous Effects of Visible Light,” Journal of the American Academy of Dermatology 84, no. 5 (2021): 1219–1231, 10.1016/j.jaad.2021.02.048. 33640508 PMC8887026

[2] P. K. Farris and G. Valacchi , “Ultraviolet Light Protection: Is It Really Enough?,” Antioxidants 11, no. 8 (2022): 1484, 10.3390/antiox11081484. 36009203 PMC9405175

[3] J. Y. Furukawa , R. M. Martinez , A. L. Morocho‐Jácome , et al., “Skin Impacts From Exposure to Ultraviolet, Visible, Infrared, and Artificial Lights ‐ a Review,” Journal of Cosmetic and Laser Therapy 23, no. 1–2 (2021): 1–7, 10.1080/14764172.2021.1950767. 34669525

[4] L. Duteil , C. Queille‐Roussel , J. P. Lacour , H. Montaudié , and T. Passeron , “Short‐Term Exposure to Blue Light Emitted by Electronic Devices Does Not Worsen Melasma,” Journal of the American Academy of Dermatology 83, no. 3 (2020): 913–914, 10.1016/j.jaad.2019.12.047. 31887321

[5] N. Garimano , T. Aguayo Frías , and D. H. González Maglio , “Beyond Ultraviolet Radiation: Immune System Modulation Through Skin Exposure to Visible Light and Infrared Radiation,” Photochemistry and Photobiology 101, no. 4 (2025): 846–857, 10.1111/php.14117. 40376965

[6] E. Yu , S. W. Oh , S. H. Park , et al., “The Pigmentation of Blue Light Is Mediated by Both Melanogenesis Activation and Autophagy Inhibition Through OPN3‐TRPV1,” Journal of Investigative Dermatology 145, no. 4 (2025): 908–918.e6, 10.1016/j.jid.2024.07.034. 39241981

[7] T. Passeron , H. W. Lim , C. L. Goh , et al., “Photoprotection According to Skin Phototype and Dermatoses: Practical Recommendations From an Expert Panel,” Journal of the European Academy of Dermatology and Venereology 35, no. 7 (2021): 1460–1469, 10.1111/jdv.17242. 33764577 PMC8252523

[8] G. Pellacani , H. W. Lim , E. Stockfleth , V. Sibaud , A. O. Brugués , and M. Saint Aroman , “Photoprotection: Current Developments and Controversies,” Journal of the European Academy of Dermatology and Venereology 38, no. Suppl. 5 (2024): 12–20, 10.1111/jdv.19677. 38924160

[9] S. Premi , S. Wallisch , C. M. Mano , et al., “Chemiexcitation of Melanin Derivatives Induces DNA Photoproducts Long After UV Exposure,” Science 347, no. 6224 (2015): 842–847, 10.1126/science.1256022. 25700512 PMC4432913

[10] A. B. Lyons , C. Trullas , I. Kohli , I. H. Hamzavi , and H. W. Lim , “Photoprotection Beyond Ultraviolet Radiation: A Review of Tinted Sunscreens,” Journal of the American Academy of Dermatology 84, no. 5 (2021): 1393–1397, 10.1016/j.jaad.2020.04.079. 32335182

[11] T. Gracia‐Cazaña , J. Aguilera , A. Navarro‐Bielsa , S. González , H. W. Lim , and Y. Gilaberte , “New Trends on Personalized Sunscreens,” Photodermatology, Photoimmunology & Photomedicine 40, no. 3 (2024): e12967, 10.1111/phpp.12967. 38616500

[12] J. A. Nichols and S. K. Katiyar , “Skin Photoprotection by Natural Polyphenols: Anti‐Inflammatory, Antioxidant and DNA Repair Mechanisms,” Archives of Dermatological Research 302, no. 2 (2009): 71–83, 10.1007/s00403-009-1001-3. 19898857 PMC2813915

[13] S. Dunaway , R. Odin , L. Zhou , L. Ji , Y. Zhang , and A. L. Kadekaro , “Natural Antioxidants: Multiple Mechanisms to Protect Skin From Solar Radiation,” Frontiers in Pharmacology 24, no. 9 (2018): 392, 10.3389/fphar.2018.00392. PMC5928335 29740318

[14] A. B. Lyons , R. Zubair , I. Kohli , et al., “Mitigating Visible Light and Long Wavelength UVA1‐Induced Effects With Topical Antioxidants,” Photochemistry and Photobiology 98, no. 2 (2022): 455–460, 10.1111/php.13525. 34549819

[15] D. Moher , A. Liberati , J. Tetzlaff , D. G. Altman , and PRISMA Group , “Preferred Reporting Items for Systematic Reviews and Meta‐Analyses: The PRISMA Statement,” International Journal of Surgery 8, no. 5 (2010): 336–341, 10.1016/j.ijsu.2010.02.007. 20171303

[16] M. Amir‐Behghadami and A. Janati , “Population, Intervention, Comparison, Outcomes and Study (PICOS) Design as a Framework to Formulate Eligibility Criteria in Systematic Reviews,” Emergency Medicine Journal 37, no. 6 (2020): 387, 10.1136/emermed-2020-209567. 32253195

[17] M. J. Page , J. E. McKenzie , P. M. Bossuyt , et al., “The PRISMA 2020 Statement: An Updated Guideline for Reporting Systematic Reviews,” BMJ 372 (2021): n71, 10.1136/bmj.n71. 33782057 PMC8005924

[18] R. Campiche , S. J. Curpen , V. Lutchmanen‐Kolanthan , et al., “Pigmentation Effects of Blue Light Irradiation on Skin and How to Protect Against Them,” International Journal of Cosmetic Science 42, no. 4 (2020): 399–406, 10.1111/ics.12637. 32478879 PMC7496068

[19] A. Mereniuk , P. Marchessault , C. Maari , C. Bolduc , and R. Bissonnette , “Topical Vitamin E Cream Does Not Prevent Visible Light‐Induced Pigmentation,” Journal of Cutaneous Medicine and Surgery 22, no. 1 (2018): 100–101, 10.1177/1203475417724440. 29309242

[20] N. Ezekwe , A. Pourang , A. B. Lyons , et al., “Evaluation of the Protection of Sunscreen Products Against Long Wavelength Ultraviolet A1 and Visible Light‐Induced Biological Effects,” Photodermatology, Photoimmunology & Photomedicine 40, no. 1 (2024): e12937, 10.1111/phpp.12937. 38069506

[21] E. Ruvolo , W. Boothby‐Shoemaker , N. Kumar , I. H. Hamzavi , H. W. Lim , and I. Kohli , “Evaluation of Efficacy of Antioxidant‐Enriched Sunscreen Prodcuts Against Long Wavelength Ultraviolet A1 and Visible Light,” International Journal of Cosmetic Science 44, no. 3 (2022): 394–402, 10.1111/ics.12785. 35587114

[22] I. Kohli , R. Shafi , P. Isedeh , et al., “The Impact of Oral Polypodium Leucotomos Extract on Ultraviolet B Response: A Human Clinical Study,” Journal of the American Academy of Dermatology 77, no. 1 (2017): 33–41.e1, 10.1016/j.jaad.2017.01.044. 28341348 PMC5730054

[23] A. Zamarrón , S. Lorrio , S. González , and Á. Juarranz , “Fernblock Prevents Dermal Cell Damage Induced by Visible and Infrared A Radiation,” International Journal of Molecular Sciences 19, no. 8 (2018): 2250, 10.3390/ijms19082250. 30071607 PMC6121512

[24] M. Portillo , M. Mataix , M. Alonso‐Juarranz , et al., “The Aqueous Extract of Polypodium Leucotomos (Fernblock) Regulates Opsin 3 and Prevents Photooxidation of Melanin Precursors on Skin Cells Exposed to Blue Light Emitted From Digital Devices,” Antioxidants 10, no. 3 (2021): 400, 10.3390/antiox10030400. 33800784 PMC7998284

[25] R. Avola , A. C. E. Graziano , G. Pannuzzo , F. Bonina , and V. Cardile , “Hydroxytyrosol From Olive Fruits Prevents Blue‐Light‐Induced Damage in Human Keratinocytes and Fibroblasts,” Journal of Cellular Physiology 234, no. 6 (2019): 9065–9076, 10.1002/jcp.27584. 30367495

[26] J. V. Freitas , H. C. Junqueira , W. K. Martins , M. S. Baptista , and L. R. Gaspar , “Antioxidant Role on the Protection of Melanocytes Against Visible Light‐Induced Photodamage,” Free Radical Biology & Medicine 131 (2019): 399–407, 10.1016/j.freeradbiomed.2018.12.028. 30590132

[27] J. I. Park , S. J. Kim , Y. J. Kim , and S. J. Lee , “Protective Role of *Caesalpinia sappan* Extract and Its Main Component Brazilin Against Blue Light‐Induced Damage in Human Fibroblasts,” Journal of Cosmetic Dermatology 21, no. 12 (2022): 7025–7034, 10.1111/jocd.15354. 36057446

[28] J. J. Cho , J. I. Chae , G. Yoon , et al., “Licochalcone A, a Natural Chalconoid Isolated From Glycyrrhiza Inflata Root, Induces Apoptosis via Sp1 and Sp1 Regulatory Proteins in Oral Squamous Cell Carcinoma,” International Journal of Oncology 45, no. 2 (2014): 667–674, 10.3892/ijo.2014.2461. 24858379

[29] T. Mann , K. Eggers , F. Rippke , et al., “High‐Energy Visible Light at Ambient Doses and Intensities Induces Oxidative Stress of Skin‐Protective Effects of the Antioxidant and Nrf2 Inducer Licochalcone A In Vitro and In Vivo,” Photodermatology, Photoimmunology & Photomedicine 36, no. 2 (2020): 135–144, 10.1111/phpp.12523. PMC7078816 31661571

[30] G. Pressi , O. Bertaiola , C. Guarnerio , et al., “In Vitro Cultured *Melissa officinalis* Cells as Effective Ingredient to Protect Skin Against Oxidative Stress, Blue Light, and Infrared Irradiations Damages,” Cosmetics 8, no. 1 (2021): 23, 10.3390/cosmetics8010023.

[31] S. Lorrio , A. Rodríguez‐Luna , P. Delgado‐Wicke , et al., “Protective Effect of the Aqueous Extract of *Deschampsia antarctica* (EDAFENCE) on Skin Cells Against Blue Light Emitted From Digital Devices,” International Journal of Molecular Sciences 21, no. 3 (2020): 988, 10.3390/ijms21030988. 32024276 PMC7038134

[32] J. Y. Park , S. H. Park , S. W. Oh , et al., “Yellow Chaste Weed and Its Components, Apigenin and Galangin, Affect Proliferation and Oxidative Stress in Blue Light‐Irradiated HaCaT Cells,” Nutrients 14, no. 6 (2022): 1217, 10.3390/nu14061217. 35334874 PMC8953766

[33] D. Son , J. S. Jun , and K. Hong , “Photoprotection Effect of Pu'er Tea and *Curcuma longa* L. Extracts Against UV and Blue Lights,” Journal of Applied Biological Chemistry 66 (2023): 106–113, 10.3839/jabc.2023.016.

[34] H. Y. Lee and J. S. Kim , “Cherry Fruit Anthocyanins Cyanidin‐3‐O‐Glucoside and Cyanidin‐3‐O‐Rutinoside Protect Against Blue Light‐Induced Cytotoxicity in HaCaT Cells,” Applied Biological Chemistry 66, no. 3 (2023): 1–12, 10.1186/s13765-023-00767-5.

[35] E. Leis Ayres , J. Dos Santos Silva , S. Eberlin , G. Facchini , C. Vasconcellos , and A. Da Costa , “Invitro Effect of Pine Bark Extract on Melanin Synthesis, Tyrosinase Activity, Production of Endothelin‐1, and PPAR in Cultured Melanocytes Exposed to Ultraviolet, Infrared, and Visible Light Radiation,” Journal of Cosmetic Dermatology 21, no. 3 (2022): 1234–1242, 10.1111/jocd.14202. 33960120

[36] T. F. Mohammad , I. Kohli , C. L. Nicholson , et al., “Oral Polypodium Leucotomos Extract and Its Impact on Visible Light‐Induced Pigmentation in Human Subjects,” Journal of Drugs in Dermatology 18, no. 12 (2019): 1198–1203. 31859468

[37] F. Wu , B. Dang , L. Hu , et al., “ *Lycium barbarum* Polysaccharide Inhibits Blue‐Light‐Induced Skin Oxidative Damage With the Involvement of Mitophagy,” Photochemistry and Photobiology 100, no. 3 (2024): 604–621, 10.1111/php.13863. 37814779

[38] C. Botta , C. Di Giorgio , A. S. Sabatier , and M. De Méo , “Genotoxicity of Visible Light (400‐800 Nm) and Photoprotection Assessment of Ectoin, L‐Ergothioneine and Mannitol and Four Sunscreens,” Journal of Photochemistry and Photobiology. B 91, no. 1 (2008): 24–34, 10.1016/j.jphotobiol.2008.01.008. 18325778

[39] F. Liebel , S. Kaur , E. Ruvolo , N. Kollias , and M. D. Southall , “Irradiation of Skin With Visible Light Induces Reactive Oxygen Species and Matrix‐Degrading Enzymes,” Journal of Investigative Dermatology 132, no. 7 (2012): 1901–1907, 10.1038/jid.2011.476. 22318388

