# Supplementary material for: Natural Agents for Preventing Skin Damage Induced by Visible Light: A Systematic Review of Preclinical and Clinical Evidence
Source: Photodermatol Photoimmunol Photomed. 2026 Aug 6;42(5):e70113. doi: 10.1111/phpp.70113 (PMC13444213; doi:10.1111/phpp.70113)
Supplement: Supplementary file 2 [file PHPP-42-e70113-s001.docx]

**Supplementary File 1.**

| Database | Search strategy | Number of results |
| --- | --- | --- |
| PubMed | ("visible light" OR "blue light" OR "high-energy visible light" OR “red light” OR “green light”)  AND ("skin damage" OR "oxidative stress" OR "inflammation" OR "photoaging" OR “photoprotection”)  AND ("natural compounds" OR "botanical extracts" OR "phytochemicals" OR "flavonoids" OR "antioxidants")  AND ("in vitro" OR "cell culture" OR "cell line" OR "human keratinocytes" OR "human fibroblasts") | 77 |
| Cochrane | ("visible light" OR "blue light" OR "high-energy visible light" OR “red light” OR “green light”)  AND ("skin damage" OR "oxidative stress" OR "inflammation" OR "photoaging" OR “photoprotection”)  AND ("natural compounds" OR "botanical extracts" OR "phytochemicals" OR "flavonoids" OR "antioxidants")  AND ("in vitro" OR "cell culture" OR "cell line" OR "human keratinocytes" OR "human fibroblasts") | 4 |
| Scopus | ("visible light" OR "blue light" OR "high-energy visible light" OR “red light” OR “green light”)  AND ("skin damage" OR "oxidative stress" OR "inflammation" OR "photoaging" OR “photoprotection”)  AND ("natural compounds" OR "botanical extracts" OR "phytochemicals" OR "flavonoids" OR "antioxidants")  AND ("in vitro" OR "cell culture" OR "cell line" OR "human keratinocytes" OR "human fibroblasts") | 192 |
| WOS | ("visible light" OR "blue light" OR "high-energy visible light" OR “red light” OR “green light”)  AND ("skin damage" OR "oxidative stress" OR "inflammation" OR "photoaging" OR “photoprotection”)  AND ("natural compounds" OR "botanical extracts" OR "phytochemicals" OR "flavonoids" OR "antioxidants")  AND ("in vitro" OR "cell culture" OR "cell line" OR "human keratinocytes" OR "human fibroblasts") | 191 |
| TOTAL |  | **464** |

**Supplementary Table S1.** Detailed literature search strategy

**Supplementary File 2.**

| Summary of findings | | |  | Certainty in evidence based on the GRADE approach | | | | | |
| --- | --- | --- | --- | --- | --- | --- | --- | --- | --- |
| Outcome | Studies (n/k) | Participants (N) | Risk of bias | | Inconsistency | Indirectness | Imprecision | Level of evidence | Importance |
| Visible light–induced pigmentation (ITA°, ΔE, PPD, melanin index) | 6/6 | 107 | Serious^1^  (-1) | | No | No | Serious^2^  (-1) | Moderate | Critical |
| Visible light–induced erythema (a*, DRS, IGA) | 4/4 | 87 | Serious^3^  (-1) | | No | No | Serious^4^  (-1) | Low | Critical |
| VL-induced molecular skin damage (CPDs, PCNA, Ki67) | 1/1 | 22 | Serious^5^  (-1) | | No | No | Very serious^6^  (-1) | Very Low | Critical |

Note: GRADE = Grading of Recommendations Assessment, Development and Evaluation.

1. Downgraded by one level due to methodological limitations in the majority of included studies, including moderate risk of bias in non-randomized designs and one study with serious risk of bias related to confounding, lack of randomization, and incomplete control of baseline pigmentation.
2. Downgraded by one level because of small overall sample size, limited number of clinical studies, and frequent absence of reported measures of variability (standard deviations or confidence intervals), precluding robust estimation of effect magnitude and formal quantitative synthesis.
3. Downgraded by one level due to methodological limitations across the included studies. One randomized trial presented *some concerns* regarding the randomization process and blinding, two non-randomized studies showed moderate risk of bias, and one study was judged at serious risk of bias due to confounding and non-randomized site allocation. In addition, protocol preregistration and full blinding of outcome assessment were frequently absent.
4. Downgraded by one level because of small overall sample size, limited number of clinical studies, and incomplete reporting of measures of variability (standard deviations or confidence intervals) for colorimetric and clinical erythema outcomes, resulting in reduced statistical precision and uncertainty regarding the magnitude of effect.
5. Downgraded by one level because the evidence is derived from a single non-randomized interventional study with serious risk of bias due to confounding, absence of random sequence generation, lack of allocation concealment, and no protocol preregistration. In addition, blinding of participants and outcome assessors was insufficiently reported.
6. Downgraded by two levels owing to very small sample size and reliance on a single clinical study, resulting in very limited statistical power and high fragility of effect estimates. Measures of variability and confidence intervals were incompletely reported, precluding robust estimation of effect magnitude.

**Supplementary Table S2.** Certainty of evidence (GRADE) for clinical outcomes.

**Supplementary File 3.**

**Supplementary figure S1.** OHAT traffic light plot for preclinical studies.
